# Pathogenicity and selective constraint on variation near splice sites

**DOI:** 10.1101/gr.238444.118

**Published:** 2019-02

**Authors:** Jenny Lord, Giuseppe Gallone, Patrick J. Short, Jeremy F. McRae, Holly Ironfield, Elizabeth H. Wynn, Sebastian S. Gerety, Liu He, Bronwyn Kerr, Diana S. Johnson, Emma McCann, Esther Kinning, Frances Flinter, I. Karen Temple, Jill Clayton-Smith, Meriel McEntagart, Sally Ann Lynch, Shelagh Joss, Sofia Douzgou, Tabib Dabir, Virginia Clowes, Vivienne P.M. McConnell, Wayne Lam, Caroline F. Wright, David R. FitzPatrick, Helen V. Firth, Jeffrey C. Barrett, Matthew E. Hurles, on behalf of the Deciphering Developmental Disorders study

**Affiliations:** 1 Wellcome Sanger Institute, Wellcome Genome Campus, Hinxton, Cambridge CB10 1SA, United Kingdom;; 2 Manchester Centre for Genomic Medicine, St. Mary's Hospital, Manchester University Hospitals NHS Foundation Trust, Manchester Academic Health Sciences Centre, Manchester M13 9WL, United Kingdom;; 3 Division of Evolution and Genomic Sciences, School of Biological Sciences, University of Manchester, Manchester M13 9NT, United Kingdom;; 4 Sheffield Clinical Genetics Service, Sheffield Children's Hospital, OPD2, Northern General Hospital, Sheffield S5 7AU, United Kingdom;; 5 Liverpool Women's Hospital Foundation Trust, Liverpool L8 7SS, United Kingdom;; 6 West of Scotland Regional Genetics Service, NHS Greater Glasgow and Clyde, Institute of Medical Genetics, Yorkhill Hospital, Glasgow G3 8SJ, United Kingdom;; 7 South East Thames Regional Genetics Centre, Guy's and St Thomas’ NHS Foundation Trust, Guy's Hospital, London SE1 9RT, United Kingdom;; 8 Faculty of Medicine, University of Southampton, Institute of Developmental Sciences, Southampton SO16 6YD, United Kingdom;; 9 Wessex Clinical Genetics Service, University Hospital Southampton, Princess Anne Hospital, Southampton SO16 5YA, United Kingdom;; 10 South West Thames Regional Genetics Centre, St. George's Healthcare NHS Trust, St. George's, University of London, London SW17 0RE, United Kingdom;; 11 Temple Street Children's Hospital, Dublin 1, Ireland;; 12 West of Scotland Regional Genetics Service, NHS Greater Glasgow and Clyde, Queen Elizabeth University Hospital, Glasgow G51 4TF, United Kingdom;; 13 Northern Ireland Regional Genetics Centre, Belfast Health and Social Care Trust, Belfast City Hospital, Belfast BT9 7AB, United Kingom;; 14 North West Thames Regional Genetics Service, London North West University Healthcare NHS Trust, Northwick Park and St. Mark's Hospitals, Harrow HA1 3UJ, United Kingdom;; 15 MRC Human Genetics Unit, MRC IGMM, University of Edinburgh, Western General Hospital, Edinburgh EH4 2XU, United Kingdom;; 16 Institute of Biomedical and Clinical Science, University of Exeter Medical School, Exeter EX2 5DW, United Kingdom;; 17 East Anglian Medical Genetics Service, Cambridge University Hospitals NHS Foundation Trust, Cambridge CB2 0QQ, United Kingdom

## Abstract

Mutations that perturb normal pre-mRNA splicing are significant contributors to human disease. We used exome sequencing data from 7833 probands with developmental disorders (DDs) and their unaffected parents, as well as more than 60,000 aggregated exomes from the Exome Aggregation Consortium, to investigate selection around the splice sites and quantify the contribution of splicing mutations to DDs. Patterns of purifying selection, a deficit of variants in highly constrained genes in healthy subjects, and excess de novo mutations in patients highlighted particular positions within and around the consensus splice site of greater functional relevance. By using mutational burden analyses in this large cohort of proband–parent trios, we could estimate in an unbiased manner the relative contributions of mutations at canonical dinucleotides (73%) and flanking noncanonical positions (27%), and calculate the positive predictive value of pathogenicity for different classes of mutations. We identified 18 patients with likely diagnostic de novo mutations in dominant DD-associated genes at noncanonical positions in splice sites. We estimate 35%–40% of pathogenic variants in noncanonical splice site positions are missing from public databases.

Pre-mRNA splicing in humans is mediated by the major and minor spliceosomes, highly dynamic, metalloenzyme complexes composed of five key small nuclear RNAs (snRNA), along with more than 100 protein components and accessory molecules (Brody and Abelson 1985; Hang et al. 2015; Scotti and Swanson 2016). Accurate recruitment and function of the spliceosome is reliant on a plethora of *cis*-acting regulatory elements encoded within the pre-mRNA itself. Although our understanding of the underlying mechanistic processes regulating splicing has greatly increased in recent years, our ability to predict whether or not a mutation will affect splicing remains limited. However, with estimates that up to 50% of monogenic disease-causing variants may affect splicing (Teraoka et al. 1999; Ars et al. 2000), a better understanding and more coherent approach to interpretation of variants affecting splicing is needed (Cartegni et al. 2002; Baralle and Buratti 2017). With a plethora of in silico splicing pathogenicity predictors available, there is little consensus on what a gold standard for splicing pathogenicity prediction would be (Houdayer et al. 2012; Jian et al. 2014b; Tang et al. 2016). Although many of these methods perform well within the canonical splice site (CSS) dinucleotides (the two highly conserved bases flanking the acceptor and donor sites), their utility for other splice relevant regions is less clear (Tang et al. 2016). In the clinical setting, often multiple algorithms and expert judgment are used to predict pathogenicity, whereas for large-scale research projects, the classification of variants is often binary, with CSS mutations typically classified as likely splice affecting, whereas mutations in other splicing regulatory components are typically overlooked (Iossifov et al. 2014; Wright et al. 2015; Deciphering Developmental Disorders Study 2017). Previous attempts to estimate the relative contribution of pathogenic variants at non-CSSs were based on collating diverse published data sets of pathogenic variants (Lewandowska 2013) or on data submitted to databases of clinically interpreted variation (Krawczak et al. 2007) and are therefore sensitive to the inherent heterogeneity and biases of such data, especially given the inevitable subjectivity involved in clinical interpretation of this class of variation. Both clinical and research interpretation of potential splice-disrupting variants lacks a robust quantitative foundation.

By using large-scale exome sequencing data from 13,750 unaffected parents recruited as part of the Deciphering Developmental Disorders (DDD) project (Wright et al. 2015) and more than 60,000 aggregated exomes from the Exome Aggregation Consortium (ExAC) (Lek et al. 2016), we explore selective constraint around splice regions across a set of 148,244 stringently defined exons well covered (median coverage >15× at both CSSs) across the DDD cohort (see Methods). Because selection is driven by a number of factors, including monogenic developmental disorders (DDs), as a complementary, disease-based approach, we analyze enrichment of de novo mutations (DNMs) in DDD probands in the same regions. We provide an unbiased, exome-wide view of the signatures of selection and the relative contribution of pathogenic splice altering mutations between the CSS and other near-splice positions.

## Results

### Signatures of purifying selection around the splice site

**Figure 1.**
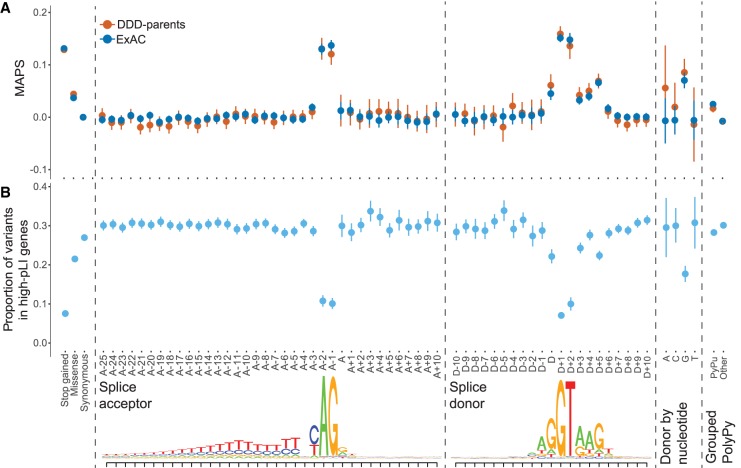
Signals of purifying selection around splice sites. (*A*) Selective constraint across splicing region in 13,750 unaffected parents of DDD probands and more than 60,000 aggregated exomes from ExAC. Mutability-adjusted proportion of singletons (MAPS) with 95% confidence intervals (CIs) shown for Ensembl's Variant Effect Predictor (VEP) annotated exonic sites, extended splice acceptor and splice donor regions, the last base of the exon, split by reference nucleotide, and grouped sites in the polypyrimidine tract (PolyPy) region, split by changes from a pyrimidine to a purine (PyPu) versus all other changes. (*B*) Proportion of variants with 95% CI in 13,750 unaffected parents of DDD probands that fall within genes with high probability of loss-of-function intolerance (pLI > 0.9) across VEP annotated exonic sites, extended splice acceptor and splice donor regions, the last base of the exon, split by reference nucleotide, and grouped sites in the PolyPy region, split by changes from a pyrimidine to a purine (PyPu) versus all other changes. *Lower* panel shows splice acceptor and splice donor consensus sequences, based on our exons of interest.

Because purifying selection acts to keep deleterious alleles rare, population variation data can be used to identify and assess the relative strengths of signals of purifying selection. To assess selective constraint acting on positions around the CSS, we used the mutability adjusted proportion of singletons (MAPS) metric (a measure for inferring the degree of selection robust to local variance in mutation rate) (Lek et al. 2016) in 13,750 unaffected parents enrolled in the DDD study as well as more than 60,000 aggregated exomes from ExAC (Fig. 1A). The canonical splice acceptor and donor dinucleotides show a clear signal of purifying selection in both data sets.

Outside of the CSS, other positions clearly show a signal of purifying selection beyond the background level, including the donor site (last base of the exon, which is particularly pronounced when the reference allele is G) (Fig. 1A), and the intronic positions proximal to the canonical donor site, peaking at the don+5 position, which shows a signal of purifying selection intermediate between missense and stop-gained variants. Although no sites within the polypyrimidine tract (PolyPy) show a signal of purifying selection individually, when these sites are grouped together (Methods) and stratified by changes from a pyrimidine to a purine (PyPu) versus all other changes, there is a clear difference between the two types of variants, with PyPu changes showing an increased signal of purifying selection compared with non-PyPu changes (bootstrap *P* < 0.001) (Fig. 1A; Supplemental Fig. S1).

### Deficit of splicing variants in highly constrained genes in healthy individuals

We also examined the distribution of variants of different classes among genes that are known to be under different levels of selective constraint. Highly constrained genes should contain fewer deleterious variants than less constrained genes. We investigated the proportion of variants observed in the 13,750 unaffected parents that fell within highly constrained genes (probability of loss-of-function [LoF] intolerance (pLI > 0.9) (Lek et al. 2016) in our splicing regions of interest (Fig. 1B). In the near-splice positions at which the highest MAPS values were seen (CSS, donor, donor+5), we also observed a stronger depletion of variants in high-pLI genes within the unaffected parents, again supporting the potential pathogenicity of variants at these positions. The proportion of parental variants in high-pLI genes also recapitulates the signals of purifying selection seen in the MAPS analyses with regard to the donor position split by reference allele (Fig. 1B) and the PolyPy region (Fig. 1B; Supplemental Fig. S1), with the lowest proportions in high-pLI genes observed for sites with the highest MAPS values.

### Assessing the significance of mutational burden for different classes of splicing mutations

**Figure 2.**
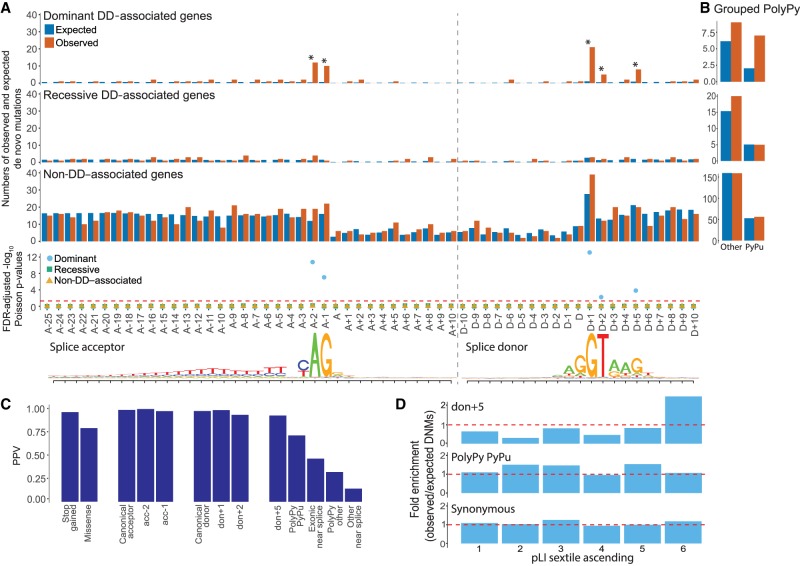
De novo mutations (DNMs) around splice sites. Enrichment of DNMs across the splicing region in 7833 DDD probands. (*A*) Numbers of observed and expected DNMs across the splicing region in known dominant and recessive DD genes, as well as in non-DD–associated genes, with FDR-corrected Poisson *P*-values. Splice acceptor and splice donor consensus sequences are shown *below*, as in Figure 1. (*B*) Aggregation of observed and expected numbers of DNMs in the PolyPy region, with changes from a pyrimidine to a purine (PyPu) and all other changes shown separately for known dominant and recessive DD genes, as well as non-DD–associated genes. (*C*) Positive predictive values (PPVs) for DNMs in dominant DD-associated genes in positions across the splicing region, as well as VEP annotated stop gained and missense changes, calculated from observed and expected numbers of DNMs. (*D*) Enrichment (observed/expected) of DNMs by gene probability of pLI split into sextiles for donor+5, pyrimidine to purine PolyPy, and synonymous sites. pLI scores encompassed by each sextile: 1 = 5.36 × 10^−91^–0.000000605, 2 = 0.000000609–0.000558185, 3 = 0.000559475–0.027905143, 4 = 0.027908298–0.377456159, 5 = 0.377491926–0.919495985, 6 = 0.91955878–1.

We identified 871 autosomal high-confidence DNMs (nonsynonymous consequences excluded) within canonical and near-splice regions of interest well covered by exome data in the 7833 probands, allowing us to test for enrichment of DNMs relative to expectations based on a trinucleotide null model of mutation rate (Samocha et al. 2014) across different sets of genes (DD-associated with dominant or recessive mechanisms, and non-DD–associated; see Methods). Across recessive DD and non-DD–associated genes, no enrichment of DNMs beyond the null expectation was observed (Fig. 2A). In dominant DD genes, a significant cumulative excess of DNMs was noted across the full splicing region (Poisson *P* = 1.33 × 10^−14^, false-discovery rate [FDR]-adjusted; fold enrichment = 3.47), which remained significant upon exclusion of the canonical dinucleotide positions (Poisson *P* [FDR-adjusted] = 0.0035, fold enrichment = 1.86). Individually, the four CSS positions each showed a significant (10- to 27-fold) excess of DNMs (Poisson *P* [FDR-adjusted], fold enrichment: acc-2 = 4.22 × 10^−12^, 26.6; acc-1 = 3.43 × 10^−8^, 16.6; don+1 = 1.33 × 10^−14^, 20.1; don+2 = 0.004, 10.0), as did the don+5 site (9.7 × 10^−5^, 9.29). The similar level of enrichment between don+5 and don+2 implies these positions harbor comparable proportions of splice disrupting mutations. No individual positions within the PolyPy region showed an individual excess of DNMs; however when the positions were considered cumulatively and split between PyPu and non-PyPu changes (Fig. 2B), an excess of DNMs was observed in the PyPu group for dominant DD genes (fold enrichment = 3.46), although this was not significant at an FDR of 5% (Poisson *P* [FDR corrected] = 0.086).

When the same analysis was performed for dominant genes in subsets of the DDD cohort with (*n* = 1417) and without (*n* = 3364) robust diagnoses from the standard diagnostic protocol, which only assesses splicing mutations at the CSS (Supplemental Fig. S2), the enrichment within the diagnosed subset was confined to the CSS (Poisson *P* [FDR-adjusted], fold enrichment: CSS = 1.33 × 10^−14^, 69.74; other positions = 0.658, 1.82), whereas in the undiagnosed subset, the opposite pattern was observed (Poisson *P* [FDR-adjusted], fold enrichment: CSS = 1, 0; other positions = 0.012, 2.21), with the don+5 site showing the greatest enrichment (16.18, Poisson *P* [FDR-adjusted] = 5.35 × 10^−5^).

These results are highly concordant with the signatures of purifying selection identified using the MAPS metric and the deficit of parental variants in high-pLI genes, providing multiple independent lines of evidence that mutations in positions outside of the CSS can disrupt normal splicing.

### Estimating positive predictive values for different classes of splice mutations

We used the fold enrichment of the numbers of observed DNMs in dominant DD genes in the DDD cohort over the number expected under the null mutation model to calculate positive predictive values (PPVs) for groupings of near-splice site positions. We compared these with PPVs for other, more commonly disease-associated variant classes within the same exons of the same genes (Fig. 2C). We observe minor differences in PPV for the individual positions of the canonical acceptor and donor sites, with don+2 showing the lowest PPV at 0.90, which is approximately the same as that for the don+5 position (0.89). Variants within the PolyPy region that change a pyrimidine for a purine have a PPV of 0.71, which is below the PPV for missense mutations (0.79) but still predicts a substantive number of pathogenic mutations arising from disruption of the PolyPy.

Despite the modest number of observed DNMs used to make these PPV estimates, we see concordance with the population-based metrics described above (for concordance with MAPS and deficit of splicing variants in high-pLI genes in unaffected parents of DDD patients, see Supplemental Fig. S3), suggesting these estimates are robust.

We looked at the distribution of observed DNMs in genes with respect to their probability of being LoF intolerant (using the pLI metric) (Fig. 2D; Lek et al. 2016). For synonymous variants, we observed no significant enrichment of DNMs in high-pLI genes. For don+5 mutations, there is a clear excess of DNMs in genes most likely to be intolerant to LoF mutations in the DDD cohort, further supporting the likely pathogenicity of mutations in these positions. For the PolyPy PyPu mutations, although there is a nominally significant enrichment of DNMs in general, this does not show a significant skew toward high-pLI genes in our cohort.

### Identifying diagnostic noncanonical splice mutations

**Figure 3.**
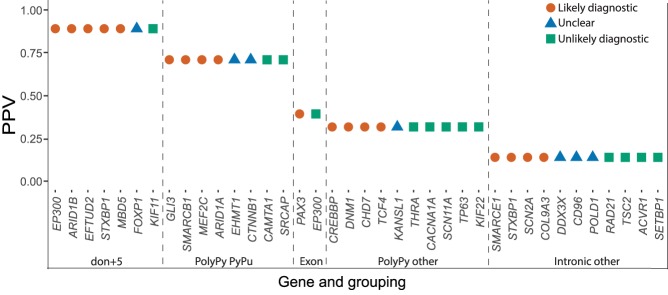
Clinical classifications of noncanonical near-splice DNMs. Relationship between clinical classifications of 38 splice region DNMs in undiagnosed DDD probands and PPVs calculated using observed and expected numbers of DNMs in 7833 probands.

After exclusion of probands with likely diagnostic protein-coding or CSS variants, 38 DNMs in our near-splice site positions of interest in dominant DD genes were identified. The clinical phenotypes of patients carrying these mutations were reviewed by a consultant clinical geneticist, blinded to the precise mutation and PPVs estimated above, and by the patient's recruiting clinician to assess the phenotypic similarity between the proband and the disorder expected from a LoF mutation in that gene. The 38 variants were classified as likely diagnostic (Table 1) or unlikely diagnostic/unknown (Supplemental Table S1), depending on the strength of phenotypic similarity. Phenotypic information for probands with likely diagnostic variants is given in Supplemental Table S2 and pathogenicity prediction scores for the SNVs in Supplemental Table S3. The clinical review resulted in 18 variants (47%) being classified as likely diagnostic, highly concordant with the number predicted from the overall PPV of noncanonical sites of 46%; moreover, a higher proportion of likely diagnostic variants were classified at sites with higher PPVs (Pearson correlation coefficient = 0.91, *P* = 0.033) (Fig. 3). With 48 CSS DNMs observed within the same exons in our probands, we estimate that 73% (95% CI: 60%–82%) of disease-causing splice-disrupting DNMs occur within the CSS, whereas 27% (95% CI: 18%–39%) are in noncanonical, near-splice positions.

**Table 1.**
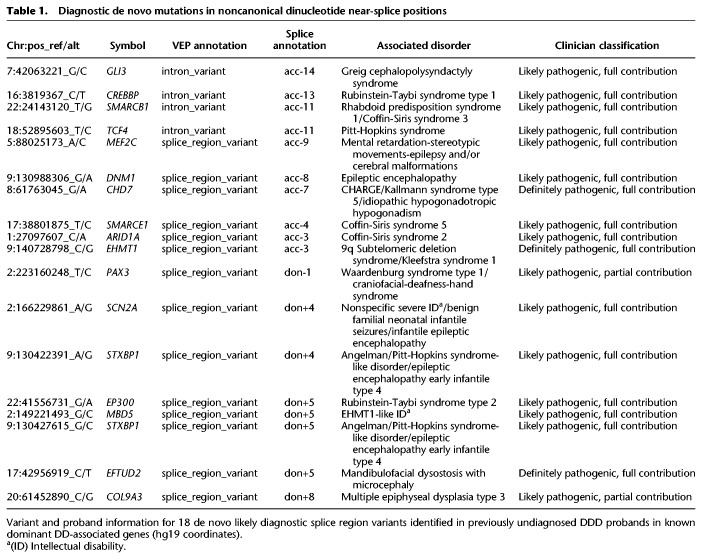
Diagnostic de novo mutations in noncanonical dinucleotide near-splice positions

Eight DNMs were selected for functional validation via a minigene vector system, including six likely diagnostic PolyPy variants, a PolyPy variant of uncertain clinical significance, and a likely diagnostic don+5 variant, in which both the phenotype of the patient and that associated with the gene (*MBD5*) are nonspecific along with two negative controls (untransmitted variants identified in unaffected parents within the same PolyPys as test variants). For six of the variants selected for validation, differences in splicing between the reference and mutant constructs were observed (Supplemental Fig. S4A,B). One of the likely diagnostic PolyPy mutations, the PolyPy mutation of uncertain significance, and both negative controls showed no difference in splicing between the reference and mutant constructs (Supplemental Fig. S4C,D).

### Assessing splicing pathogenicity prediction tools

The population genetic metrics of purifying selection and mutation enrichment metric for pathogenicity that we have derived provide an orthogonal approach to assessing the accuracy of splicing pathogenicity prediction tools, compared with the standard approach of assessing classification accuracy for clinically interpreted variants. We assessed four splicing pathogenicity prediction tools: two recently published genome-wide ensemble learning methods—AdaBoost and randomForest, Spidex (based on deep learning trained on RNA sequencing data), and the longer-standing, widely used MaxEntScan (MES) (Yeo and Burge 2004; Jian et al. 2014a; Xiong et al. 2015).

**Figure 4.**
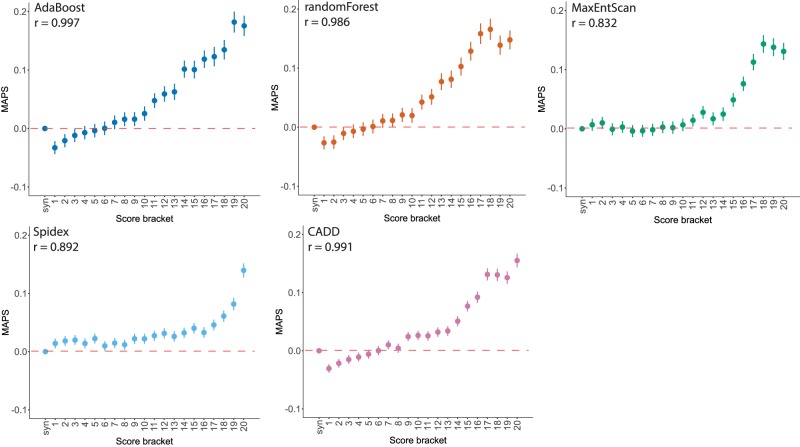
Selective constraint and pathogenicity scores. MAPS, with 95% CI, calculated for pathogenicity score brackets (least to most severe) in 13,750 unaffected parents from the DDD project, with Spearman's rank correlation coefficient.

**Figure 5.**
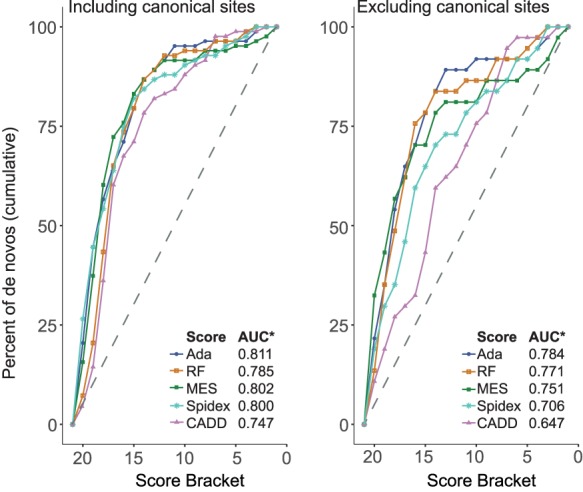
Pathogenicity scores for observed near-splice site DNMs. Cumulative percentage of DNMs in known dominant DD genes with decreasing pathogenicity score bracket, shown with canonical splice site positions included (*left*) and excluded (*right*). (AUC*) area under curve.

We divided the scores from each prediction tool, plus CADD (Kircher et al. 2014), into 20 equal-sized bins to facilitate cross-method comparability. We calculated the MAPS for each bin of each of the scoring metrics for the splicing variants observed in the 13,750 DDD unaffected parents, and saw a strong positive correlation between the pathogenicity metric and MAPS for all tools (Fig. 4). AdaBoost had the highest absolute MAPS value for the top-scoring bin, suggesting that it is best able to identify variants under the strongest purifying selection. The proportion of variants in the unaffected parents falling in genes with pLI > 0.9 broadly recapitulates this pattern, with fewer variants in high-pLI genes in the highest scoring brackets for all metrics (Supplemental Fig. S5). We then looked at the distribution of scores for each tool for the 83 splicing DNMs observed in DDD probands in autosomal dominant DD-associated genes that were covered by all five scoring systems to compare the performance of the metrics on mutations more likely to have a deleterious impact on splicing with the expectation that these potentially damaging variants would be scored highly by the metrics, giving high values of area under the curve (AUC) (Fig. 5). Again, all metrics performed well, with the majority of DNMs being classified in the most deleterious score brackets. Here AdaBoost gave the highest AUC value, suggesting it weighted these likely damaging variants as more deleterious than the other metrics comparatively. When CSS positions were removed from the analysis, AdaBoost remained the tool with the highest AUC. The largest reduction in the AUC metric was seen for Spidex and CADD, indicating these tools may be the least informative for positions outside of the CSS. Upon removal of the CSS positions from the analyses of MAPS and deficit of parental variants in high-pLI genes, similar results were revealed, with the highest AdaBoost scores retaining strong signals of purifying selection but a marked reduction in signal from the highest Spidex scores (Supplemental Figs. S6, S7).

Taken together, these data show a strong relationship between the considered splicing pathogenicity scoring systems and the general landscape of purifying selection on splicing control but show that the utility of these systems in identifying likely diagnostic variants is limited outside of the CSS.

## Discussion

Our study represents a large, unbiased exploration of the perturbation of splicing by genetic variation in near-splice regions, with complementary signals of selection observed through two different population-based analyses. Selection can be driven by many factors, including monogenic disease resulting in reduced reproductive fitness. DDs represent the largest single class of monogenic diseases. With a clear enrichment of DNMs shown by previous studies of DDs (Deciphering Developmental Disorders Study 2017), analysis of such DNMs provided an independent, disease-based approach, complementary to our population-based analyses. Concordance in results from the different approaches indicates the robustness of our conclusions.

Our analyses, taken together, suggest the pathogenic contribution of noncanonical splice positions has been underappreciated. We estimate that ∼27% (95% CI: 18%–39%) of splice disrupting pathogenic mutations within the DDD cohort are in noncanonical positions. In sites with pathogenic or likely pathogenic clinical significance in ClinVar (Landrum et al. 2016) overlapping with our splicing positions of interest (and with nonsynonymous consequences removed), we found 83.5% of variants fell within canonical positions, with just 16.5% in noncanonical positions. When adjusted for number of submissions as a proxy for allele count, this figure was 17.5%, perhaps indicative that recurrence strengthens evidence of pathogenicity. Both of these values are significantly below our estimate of 27% (Fisher's exact test *P* = 1.22 × 10^−15^ and *P* < 2.2 × 10^−16^, respectively), suggesting underascertainment of noncanonical splicing variants by ∼35%–40% in clinical databases, despite a growing understanding of the importance of such sites in splicing regulation (Kircher et al. 2014; Ferreira et al. 2016; Soukarieh et al. 2016; Cummings et al. 2017; Ito et al. 2017; Soemedi et al. 2017; Ke et al. 2018).

Estimates of the relative contribution of canonical and non-CSS mutations are sparse in the literature. These estimates are also typically based on clinically interpreted variants and so are likely to be biased by the accuracy of current clinical practices. When comparing canonical and noncanonical mutations within the Human Gene Mutation Database (HGMD), based on variation described in publications, Krawczak et al. (2007) stated canonical mutations accounted for 64% of mutations at donor sites and 77.4% of mutations at acceptor sites, giving an estimated noncanonical contribution of ∼30% overall (consistent with our data), although data taken from Caminsky et al. (2014) put this estimate at ∼43% (above our upper bound). These values are closer to our 27% estimate than to the ClinVar proportion of ∼17%, despite our approach focusing on DNMs and dominant disorders, whereas the other two studies did not discriminate on mode of inheritance and included recessive disorders, which can also be caused by noncanonical splicing mutations (Basel-Vanagaite et al. 2013; Brunham et al. 2015) and exonic variants. Our findings highlight the complementarity of assessing the clinical importance of noncanonical splice variants both through the traditional approach based on clinically interpreted variation accrued through diagnostic practice and through unbiased approaches that leverage population variation and unbiased models of germline mutation.

Our analysis of noncanonical splice position mutations did not include exonic missense variants (Teraoka et al. 1999; Ars et al. 2000; Krawczak et al. 2007) nor did it explicitly include branchpoints (Maslen et al. 1997; Di Leo et al. 2004; Crotti et al. 2009; Aten et al. 2013), splicing enhancers and suppressors (Lorson et al. 1999; Liu et al. 2001), or deep intronic mutations (Cummings et al. 2017; Vaz-Drago et al. 2017). Detecting splice disrupting variants at these sites is even more challenging, as despite recent efforts (Corvelo et al. 2010; Wang and Wang 2014; Mercer et al. 2015; Badr et al. 2016; Taggart et al. 2017), comprehensive catalogs of all branchpoints and exonic and intronic splicing enhancers and silencers are currently unavailable, algorithms that predict the impact of mutations at such sites are not highly accurate, and some of these sites are not covered by exome sequencing (the greater utilization of whole-genome sequencing will allow greater opportunity to find and assess the contributions of more distal splice disrupting variants). As such, our estimate of the contribution of noncanonical splicing position mutations is likely to be a lower bound. Thus our estimate of 35%–40% underascertainment in clinical databases may be conservative, and the true extent of missed diagnoses may be even higher.

The size of the available data sets determines our power to detect signals of selection and enrichment of DNMs; so although we could show signal at splice-important noncanonical positions, there may be other positions with more subtle signals of selection that we lacked the power to detect. Another limiting factor for such analyses is the specificity with which we can identify splicing related sequences. For splice sites themselves, well-curated resources of intron–exon junctions exist (e.g., from GENCODE), giving a high degree of confidence that what we are assessing is indeed near-splice sequence. For exonic and intronic splicing enhancers and suppressors, although attempts at comprehensive identification have been made (Fairbrother et al. 2002; Zhang and Chasin 2004; Goren et al. 2006; Ke et al. 2011), there is little concordance between available resources (Cáceres and Hurst 2013), meaning nonenhancer/suppressor sequence would almost certainly be included in analyses, limiting power to detect any signal. Collation of yet larger data sets and a greater understanding of other splicing elements will help to identify these sites, using the same methodology applied here.

The nature of many DDs makes obtaining RNA samples from relevant tissues of patients (i.e., neural tissue) acutely problematic, so we investigated the effects on splicing of several of the potentially diagnostic DNMs using a minigene vector system. We were able to show changes to splicing for five out of six likely diagnostic PolyPy variants as well as the likely diagnostic don+5 variant, supporting the clinical interpretation based on clinical phenotype. We did not observe an effect on splicing for one likely diagnostic PolyPy variant and for one PolyPy variant of uncertain clinical significance. Although the accuracy of minigene assays compared with patient RNA is generally high (Bonnet et al. 2008; Thery et al. 2011; van der Klift et al. 2015), known limitations of the system (e.g., lack of full endogenous genetic context [Baralle et al. 2006; Sangermano et al. 2018] and sensitivity to cell type used [Lastella et al. 2006]) mean we cannot definitively state that the effects seen in the minigene assay would be the same in the full genetic, developmental, and cellular context within the patient.

We envisage that greater appreciation of the importance of near-splice site mutations will increase diagnostic yields, as well as provide increased power for the detection of new genetic associations, both within the field of rare disease and beyond. We highlight two challenges to improving detection of pathogenic non-CSS mutations.

First, many commonly used in silico tools for annotating the likely functional impact of variants do not discriminate between different non-CSS positions with very different likelihoods of being pathogenic. Moreover, commonly used annotation tools differ in the ways in which variants are annotated, with splicing variants displaying the highest level of disagreement between tools (McCarthy et al. 2014). This highlights the need for a more consistent and evidence-based annotation of splicing variants. Of the positions shown in our analyses to be most damaging, don+5 sites are annotated by the Variant Effect Predictor (VEP) (McLaren et al. 2016) and SnpEff (Cingolani et al. 2012) as “splice_region_variant,” whereas most positions of the PolyPy are annotated as intronic so are potentially easily overlooked. With Annovar's (Wang et al. 2010) default settings, only the CSSs are flagged as splicing variants, although with both Annovar and SnpEff, the user can optionally extend the region to be annotated as splice variants. We note that Ensembl has recently implemented a VEP plugin that allows greater granularity in splice region annotation (https://github.com/Ensembl/VEP_plugins/blob/release/88/SpliceRegion.pm), including annotating the don+5 and other near-donor positions, as well as the PolyPy region. This type of increased granularity of splicing annotation should facilitate consideration of these variants in future studies.

Second, current tools that predict the pathogenicity of non-CSS mutations have limited accuracy, and it is not clear how to translate the scores that they output into a likelihood of pathogenicity. The quantitative framework that we introduced here of estimating PPVs for different classes of mutations by comparing the number of observed mutations to the number expected under a well-calibrated null model of germline mutation has much more direct relevance to clinical interpretation, although the interpretation of specific DNMs still proves problematic, particularly for DNMs in sites of intermediate PPV. We propose that the scores generated by such splicing prediction tools could be calibrated by performing analogous analyses of mutation enrichment to estimate PPVs for different bins of scores. As the size of trio-based cohorts increases, the accuracy of calibration will improve.

In summary, our results show a significant contribution of noncanonical splicing mutations to the genetic landscape of DDs, a finding that is highly likely to be recapitulated across other monogenic disorders and contexts. We show the importance of noncanonical positions (particularly the don+5 site and pyrimidine-removing mutations in the PolyPy region). These inferences are supported both by population genetic investigations of purifying selection and by a disease-based approach, considering the burden of DNMs in approximately 8000 children with severe DDs. Mutations at some noncanonical splicing positions convey a risk of disease similar to that of protein truncating and missense mutations but are underrepresented in existing databases of disease-causing variants.

## Methods

### Cohort and sequencing

For the full description of cohort and analytical methodology, see previous DDD publications (Deciphering Developmental Disorders Study 2015, 2017). Briefly, 7833 patients with severe, undiagnosed DDs were recruited to the DDD study from 24 clinical genetics centers from across the United Kingdom and Ireland. Whole-exome sequencing was conducted on the proband and both parents, with exome capture using SureSelect RNA baits (Agilent human all-exon V3 plus with custom ELID C0338371 and Agilent human all-exon V5 plus with custom ELID C0338371) and sequencing using 75-bp paired-end reads using Illumina's HiSeq. Mapping was conducted to GRCh37 using the Burrows–Wheeler aligner (BWA; v0.59) (Li and Durbin 2009), and variant identification was conducted using the Genome Analysis Toolkit (GATK; v3.5.0) (McKenna et al. 2010). Realigning to GRCh38 should not affect the conclusions of this work, as only high-confidence intron–exon boundaries were used in the analyses. These were taken from GENCODE v19 (GRCh37) but filtered to exclude a small subset of exons that no longer met our stringent criteria in GENCODE v22 (GRCh38), as described below. Variant annotation was conducted with Ensembl's VEP (https://www.ensembl.org/info/docs/tools/vep/index.html), using Ensembl gene build 76 (McLaren et al. 2016). DNMs were identified using DeNovoGear (v0.54) (Ramu et al. 2013) and filtered using an in house pipeline, denovoFilter, developed by Jeremy F. McRae (Deciphering Developmental Disorders Study 2017; https://github.com/jeremymcrae/denovoFilter). Exome sequencing and phenotype data are accessible via the European Genome-Phenome Archive (EGA) under accession number EGAS00001000775 (https://www.ebi.ac.uk/ega/studies/EGAS00001000775).

### Defining exons of interest

We took exons from GENCODE v19 (https://www.gencodegenes.org/human/release_19.html) that met the following criteria: annotation_type = “exon,” gene_type = “protein_coding,” gene_ status = “KNOWN,” transcript_type = “protein_coding,” transcript_ status = “KNOWN,” annotation != “level 3” (automated annotation), and tag = “CCDS,” “appris_principal,” “appris_candidate_ longest,” “appris_candidate,” or “exp_conf” (*n* = 255,812 exons) (Harrow et al. 2012). We removed a small subset of exons that no longer met these criteria in the more recent, GRCh38-based GENCODE v22 release (leaving 253,275 exons). We removed any exons in which the median coverage at the canonical acceptor or donor positions was <15× in two sets of DDD data that used different exon capture methods (Agilent human all-exon V3 plus with custom ELID C0338371 and Agilent human all-exon V5 plus with custom ELID C0338371); 148,244 exons passed these criteria.

We annotated individual genomic positions relative to the acceptor and donor sites, removing any exons <14 bp and any positions that had multiple potential annotations. At the acceptor end, we considered 25 bp of intronic sequence (acc-25 to acc-1) and 11-bp exonic sequence (acc to acc+10). At the donor end, we considered 10 bp of intronic sequence (don+1 to don+10) and 11-bp exonic sequence (don to don-10). This yielded approximately 6.9 million near-splice positions of interest.

The reference nucleotide composition at each position of the splicing region of interest was calculated using all sites, and a weighted position weight matrix graph was generated using the seqLogo package via Bioconductor (Huber et al. 2015; https://bioconductor.org/packages/release/bioc/html/seqLogo.html) in R (version 3.1.3) (R Core Team 2018).

We define the PolyPy region as acc-3 and acc-5 to acc-17 based on pyrimidine content >70% in our exons of interest. We assess changes from a pyrimidine to a purine (PyPu), adjusting for the strand containing the exon.

### MAPS

In 13,750 unaffected parents enrolled as part of the DDD study, as well as more than 60,000 aggregated exomes from ExAC v0.3.1 (http://exac.broadinstitute.org/), we calculated the MAPS metric (Lek et al. 2016) using code developed in house by Patrick J. Short (Short et al. 2018; https://github.com/pjshort/dddMAPS). The MAPS metric is based on the principle that negative selection acts to keep deleterious variation rare at a population level, but more mutable sequence contexts can contain variants that appear more common because of recent recurrent mutational events, so the metric corrects frequencies based on local sequence context using synonymous mutations. Only relevant ExAC sites with “PASS” in the VCF “FILTER” column were counted, and ExAC and DDD variants were filtered for FisherStrand (FS) <10. MAPS was calculated for all SNVs overlapping our splice positions of interest (201,587 near-splice variants for DDD, and 678,241 for ExAC), the last base of the exon split by reference nucleotide (2109 variants for DDD, 6325 for ExAC), and the PolyPy split by PyPu (15,847 variants for DDD, 58,762 for ExAC) versus all other PolyPy changes (52,300 variants for DDD, 175,287 for ExAC), as well as VEP (McLaren et al. 2016) ascertained synonymous (580,066 variants for DDD, 1,513,758 for ExAC), missense (1,125,167 variants for DDD, 2,786,533 for ExAC) and stop-gained (25,863 variants for DDD, 78,496 for ExAC) sites across autosomal regions. To establish whether the MAPS metric was significantly different between PolyPy PyPu versus all other PolyPy changes, a bootstrap resampling method was run with 1000 iterations.

### Parental variants in high-pLI genes

We annotated all variant sites used in the MAPS calculations above in the 13,750 DDD parents with the gene in which the variant fell and the pLI score of that gene and calculated the proportion of these sites that fell within genes with high pLI scores (more than 0.9) (Lek et al. 2016).

### DNMs

DNMs were identified using DeNovoGear (Ramu et al. 2013) as previously described in McRae et al. 2017 (Deciphering Developmental Disorders Study 2017), and a stringent confidence threshold (posterior probability greater than 0.8) was applied. We used triplet-based mutation rates (Samocha et al. 2014) for each potential single-nucleotide change across our splicing regions of interest to calculate the expected number of DNMs across autosomes in the 7833 probands. Expected values were adjusted for depth of sequencing coverage less than 50 to account for poorer ascertainment of variants in low-coverage regions (exon depth <1, exp × 0.119; exon depth >1 and <50, exp× (0.119+0.204 × log(depth))). The values used for this correction are based on the relationship between observed and expected synonymous DNMs at different levels of coverage. We stratified this analysis into known dominant, known recessive and non-DD–associated genes using the DDG2P gene list (http://www.ebi.ac.uk/gene2phenotype), downloaded in January 2016. Genes with recessive and dominant modes of inheritance were restricted to the recessive list (see Supplemental Table S4). Observed and expected numbers of DNMs were also calculated in subsets of the DDD probands with confident diagnoses (individuals with a reported variant classed as pathogenic or likely pathogenic by the referring clinician) and those lacking a potential diagnosis (diagnosed *n* = 1417, undiagnosed *n* = 3364, with the remainder of the cohort having possible or uncertain diagnostic states, as of January 2018). We used the Poisson test (using R's poisson.test, with two-sided alternative hypothesis) to examine differences in the observed and expected values and used a 5% FDR correction to control for multiple testing using the p.adjust R package (method = fdr) across all tests (R v3.1.3) (R Core Team 2018).

PPVs were calculated ((observed − expected)/observed) for CSS positions, combined and individually; don+5 sites; PolyPy PyPu; PolyPy other; other near splice exonic and intronic variants; and VEP defined missense and stop gained mutations.

We divided our exons into sextiles based on the pLI (Lek et al. 2016) of the gene to which they belong and calculated the observed and expected number of DNMs in each sextile for don+5, PolyPy PyPu, and synonymous variants (as above) to see if the enrichment of don+5 and PolyPy PyPu changes was concentrated in genes more likely to be intolerant of LoF mutations.

### Potential diagnostic variants

DNMs overlapping with our near-splice positions of interest within dominant DDG2P genes were identified in DDD probands lacking a potential explanatory variant (December 2016, *n* = 5907). The Human Phenotype Ontology (HPO; http://compbio.charite.de/hpoweb/)–encoded (Köhler et al. 2017) phenotypes of the probands were assessed by consultant clinical geneticist Helen V. Firth, along with the patient's recruiting clinician, and were compared with the known clinical presentation of individuals with LoF mutations within those genes, classifying each variant as likely diagnostic, unlikely diagnostic, or unsure, depending on the strength of similarity between the proband and the disorder, as well as the specificity of the phenotype. The relationship between our PPVs and the proportion of clinical diagnoses in each class of near-splice mutation was assessed using Pearson's product-moment correlation using the cor.test function in R (version 3.4.4) (R Core Team 2018).

The proportion of CSS to non-CSS splicing diagnoses was calculated, along with 95% CIs, based on 18 non-CSS diagnoses and 48 CSS diagnoses in the same regions using the prop.test package in R (version 3.4.4) (R Core Team 2018).

### Validation of putative splicing variants

Eight variants were selected for validation via a minigene vector system. These comprised six likely diagnostic variants from the PolyPy, a PolyPy variant of uncertain clinical significance, and a likely diagnostic don+5 variant. Additionally, two untransmitted variants identified in unaffected parents within the same PolyPys as test variants were selected as negative controls. Details of all variants selected for validation are shown in Supplemental Table S5.

### Cloning splicing vectors

The minigene splice assay vector was adapted from that used in Singh et al. (2016), by replacing intron 1 with the first intron from the rat insulin 2 gene (*Ins2*; Rnor_6.0 Chr 1: 215,857,148–215,857,695). To generate individual assay vectors, either the 5′-most 231 bp (for the don+5 variant) or the 3′-most 274 bp (for PolyPy variants) of this vector was replaced with the appropriate endogenous intronic sequence encompassing the DNM of interest (Supplemental Fig. S4A,B), as described below. Between 114- and 202-bp flanking endogenous intronic sequence was included, along with 6-bp local exonic sequence from the gene of interest.

First, proband genotypes (Supplemental Table S5) were verified by capillary sequencing of genomic PCR products (Supplemental Table S6). Genomic regions containing the reference and alternate sequences were then either amplified by nested PCR, generated by site-directed mutagenesis, or generated using gene synthesis (IDT). These fragments were subcloned by Gibson assembly (NEB) into our minigene vector (Supplemental Tables S7, S8). The regions assayed in our vectors are detailed by genomic coordinates in Supplemental Table S5.

### In vitro splicing assay

HeLa cells were seeded into 12-well plates at a density of 160,000 cells per well, grown for 24 h, and transfected with 1 µg of plasmid vector using Lipofectamine 3000 (Invitrogen). All transfections were performed in duplicate and cultured for 48 h. HeLa cells were cultured in DMEM (10% FCS + 1% pen/strep) at 37°C in a humidified incubator. Total RNA was extracted using a micro RNeasy Qiagen kit and mRNA converted into cDNA using SuperScript IV (Invitrogen). RT-PCR was performed using primers designed to span from exon 1 to exon 2, exon 2 to exon 3, and exon 1 to exon 3 and amplified on a thermocycler for either 25 or 35 cycles (Supplemental Table S9). Amplicons were capillary sequenced (GATC). For amplicons showing more than one splice variant (mixed capillary traces, for *CHD7*-Alt and *MBD5*-Alt), we cloned the PCR amplicons (zero blunt PCR cloning kit, Invitrogen) and sequenced individual colonies by capillary sequencing to identify the splice variants present (Supplemental Table S10).

Chromatograms were generated in R (R Core Team 2018) from .ab1 files using the sangerseqR (Hill et al. 2014) package via Bioconductor (Huber et al. 2015; http://bioconductor.org/packages/release/bioc/html/sangerseqR.html, R v3.1.3), and likely consequences on the protein primary structure were generated using reference and alternative RNA sequences with the ExPASy nucleotide sequence translation tool (Artimo et al. 2012; https://web.expasy.org/translate/).

### Splicing pathogenicity scores

Because our region of interest spanned more than 6 million individual positions, each with three potential single-nucleotide changes, we were restricted in the choice of splicing pathogenicity prediction tools we could use, as many function primarily through a low-throughput web interface model. We identified three resources recently published that provide “genome-wide” splicing pathogenicity scores. Two methods, dbscSNV's AdaBoost and randomForest are based on ensemble learning combining predictions from multiple other splice prediction tools, as well as conservation and CADD scores (Jian et al. 2014a). The targeted region at the acceptor end spans 14 bases (12 intronic, two exonic) and at the donor end spans 11 bases (eight intronic, three exonic). Spidex uses deep-learning methods trained on RNA sequencing data to estimate the consequence of variants on the “percent spliced in” of an exon relative to the reference sequence (Xiong et al. 2015). Spidex scores positions up to 300 bp from intron/exon boundaries, so it provides greater coverage of our splicing region of interest. We also used the longer standing and widely used MES (Yeo and Burge 2004), for which Perl scripts were available, allowing the tool to be run locally for all alternative alleles of all positions of interest. The metric used for MES was the percentage difference between the scores for the reference and alternative alleles, with the greatest reduction in score classed as most pathogenic. All sites were also scored with CADD (Kircher et al. 2014).

To allow cross-tool comparison, we ordered positions by increasing pathogenicity from each metric and split positions into 20 brackets such that the cumulative triplet-based mutation rate for all variants in each bracket was equal and the 20th bracket contained the positions with the most pathogenic scores. We calculated MAPS and the proportion of parental variants falling in high-pLI genes for each bracket for all five metrics, as above, and looked at the number of DNMs in known dominant genes that fell in each bracket for the five metrics. Each of these analyses was conducted including and excluding CSS dinucleotide positions.

### Splice region variants in the ClinVar database

We extracted all ClinVar (Landrum et al. 2016; https://www.ncbi.nlm.nih.gov/clinvar/) variants using the UCSC Table Browser (Karolchik et al. 2004) on February 05, 2017, and matched these against our splicing positions of interest, removing exonic sites with nonsynonymous consequences. This resulted in 3603 positions with clinical significance recorded as pathogenic or likely pathogenic. We calculated the ratio of canonical to noncanonical splice positions within these data. Because each variant is present in these data only once, we adjusted for this by using number of submissions as a proxy for allele count and calculated the ratio of canonical to noncanonical variants. Differences between these observed values and our expectations, based on 27% of splice affecting mutations being in noncanonical positions, were assessed using Fisher's exact test (R v3.1.3) (R Core Team 2018).

### Software availability

Code and data to reproduce the analyses within this paper are available in the Supplemental code and figures, as well as on GitHub (https://github.com/JLord86/DDD_Splicing).

## Competing interest statement

M.E.H. is a cofounder of, consultant to, and holds shares in Congenica, a genetics diagnostic company.

## Supplementary Material

Supplemental Material

## References

[GR238444LORC1] Ars E, Serra E, Garcia J, Kruyer H, Gaona A, Lazaro C, Estivill X. 2000 Mutations affecting mRNA splicing are the most common molecular defects in patients with neurofibromatosis type 1. Hum Mol Genet 9: 237–247. 10.1093/hmg/9.2.237 10607834

[GR238444LORC2] Artimo P, Jonnalagedda M, Arnold K, Baratin D, Csardi G, de Castro E, Duvaud S, Flegel V, Fortier A, Gasteiger E, 2012 ExPASy: SIB bioinformatics resource portal. Nucleic Acids Res 40: W597–W603. 10.1093/nar/gks400 22661580 PMC3394269

[GR238444LORC3] Aten E, Sun Y, Almomani R, Santen GW, Messemaker T, Maas SM, Breuning MH, den Dunnen JT. 2013 Exome sequencing identifies a branch point variant in Aarskog–Scott syndrome. Hum Mutat 34: 430–434. 10.1002/humu.22252 23169394

[GR238444LORC4] Badr E, ElHefnawi M, Heath LS. 2016 Computational identification of tissue-specific splicing regulatory elements in human genes from RNA-Seq data. PLoS One 11: e0166978 10.1371/journal.pone.0166978 27861625 PMC5115852

[GR238444LORC5] Baralle D, Buratti E. 2017 RNA splicing in human disease and in the clinic. Clin Sci (Lond) 131: 355–368. 10.1042/CS20160211 28202748

[GR238444LORC6] Baralle M, Skoko N, Knezevich A, De Conti L, Motti D, Bhuvanagiri M, Baralle D, Buratti E, Baralle FE. 2006 *NF1* mRNA biogenesis: effect of the genomic milieu in splicing regulation of the *NF1* exon 37 region. FEBS Lett 580: 4449–4456. 10.1016/j.febslet.2006.07.018 16870183

[GR238444LORC7] Basel-Vanagaite L, Hershkovitz T, Heyman E, Raspall-Chaure M, Kakar N, Smirin-Yosef P, Vila-Pueyo M, Kornreich L, Thiele H, Bode H, 2013 Biallelic *SZT2* mutations cause infantile encephalopathy with epilepsy and dysmorphic corpus callosum. Am J Hum Genet 93: 524–529. 10.1016/j.ajhg.2013.07.005 23932106 PMC3769928

[GR238444LORC8] Bonnet C, Krieger S, Vezain M, Rousselin A, Tournier I, Martins A, Berthet P, Chevrier A, Dugast C, Layet V, 2008 Screening *BRCA1* and *BRCA2* unclassified variants for splicing mutations using reverse transcription PCR on patient RNA and an ex vivo assay based on a splicing reporter minigene. J Med Genet 45: 438–446. 10.1136/jmg.2007.056895 18424508

[GR238444LORC9] Brody E, Abelson J. 1985 The “spliceosome”: Yeast pre-messenger RNA associates with a 40S complex in a splicing-dependent reaction. Science 228: 963–967. 10.1126/science.3890181 3890181

[GR238444LORC10] Brunham LR, Kang MH, Van Karnebeek C, Sadananda SN, Collins JA, Zhang LH, Sayson B, Miao F, Stockler S, Frohlich J, 2015 Clinical, biochemical, and molecular characterization of novel mutations in *ABCA1* in families with Tangier disease. JIMD Rep 18: 51–62. 10.1007/8904_2014_348 25308558 PMC4361929

[GR238444LORC11] Cáceres EF, Hurst LD. 2013 The evolution, impact and properties of exonic splice enhancers. Genome Biol 14: R143 10.1186/gb-2013-14-12-r143 24359918 PMC4054783

[GR238444LORC12] Caminsky N, Mucaki EJ, Rogan PK. 2014 Interpretation of mRNA splicing mutations in genetic disease: review of the literature and guidelines for information-theoretical analysis. F1000Res 3: 282 10.12688/f1000research.5654.1 25717368 PMC4329672

[GR238444LORC13] Cartegni L, Chew SL, Krainer AR. 2002 Listening to silence and understanding nonsense: exonic mutations that affect splicing. Nat Rev Genet 3: 285–298. 10.1038/nrg775 11967553

[GR238444LORC14] Cingolani P, Platts A, Wang le L, Coon M, Nguyen T, Wang L, Land SJ, Lu X, Ruden DM. 2012 A program for annotating and predicting the effects of single nucleotide polymorphisms, SnpEff: SNPs in the genome of *Drosophila melanogaster* strain *w*^1118^; *iso*-2; *iso*-3. Fly (Austin) 6: 80–92. 10.4161/fly.19695 22728672 PMC3679285

[GR238444LORC15] Corvelo A, Hallegger M, Smith CW, Eyras E. 2010 Genome-wide association between branch point properties and alternative splicing. PLoS Comput Biol 6: e1001016 10.1371/journal.pcbi.1001016 21124863 PMC2991248

[GR238444LORC16] Crotti L, Lewandowska MA, Schwartz PJ, Insolia R, Pedrazzini M, Bussani E, Dagradi F, George AL Jr, Pagani F. 2009 A *KCNH2* branch point mutation causing aberrant splicing contributes to an explanation of genotype-negative long QT syndrome. Heart Rhythm 6: 212–218. 10.1016/j.hrthm.2008.10.044 19187913

[GR238444LORC17] Cummings BB, Marshall JL, Tukiainen T, Lek M, Donkervoort S, Foley AR, Bolduc V, Waddell LB, Sandaradura SA, O'Grady GL, 2017 Improving genetic diagnosis in Mendelian disease with transcriptome sequencing. Sci Transl Med 9: eaal5209 10.1126/scitranslmed.aal5209 28424332 PMC5548421

[GR238444LORC18] Deciphering Developmental Disorders Study. 2015 Large-scale discovery of novel genetic causes of developmental disorders. Nature 519: 223–228. 10.1038/nature14135 25533962 PMC5955210

[GR238444LORC19] Deciphering Developmental Disorders Study. 2017 Prevalence and architecture of *de novo* mutations in developmental disorders. Nature 542: 433–438. 10.1038/nature21062 28135719 PMC6016744

[GR238444LORC20] Di Leo E, Panico F, Tarugi P, Battisti C, Federico A, Calandra S. 2004 A point mutation in the lariat branch point of intron 6 of *NPC1* as the cause of abnormal pre-mRNA splicing in Niemann-Pick type C disease. Hum Mutat 24: 440 10.1002/humu.9287 15459971

[GR238444LORC21] Fairbrother WG, Yeh RF, Sharp PA, Burge CB. 2002 Predictive identification of exonic splicing enhancers in human genes. Science 297: 1007–1013. 10.1126/science.1073774 12114529

[GR238444LORC22] Ferreira PG, Oti M, Barann M, Wieland T, Ezquina S, Friedlander MR, Rivas MA, Esteve-Codina A, The GEUVADIS Consortium, Rosenstiel P, 2016 Sequence variation between 462 human individuals fine-tunes functional sites of RNA processing. Sci Rep 6: 32406 10.1038/srep32406 27617755 PMC5019111

[GR238444LORC23] Goren A, Ram O, Amit M, Keren H, Lev-Maor G, Vig I, Pupko T, Ast G. 2006 Comparative analysis identifies exonic splicing regulatory sequences: the complex definition of enhancers and silencers. Mol Cell 22: 769–781. 10.1016/j.molcel.2006.05.008 16793546

[GR238444LORC24] Hang J, Wan R, Yan C, Shi Y. 2015 Structural basis of pre-mRNA splicing. Science 349: 1191–1198. 10.1126/science.aac8159 26292705

[GR238444LORC25] Harrow J, Frankish A, Gonzalez JM, Tapanari E, Diekhans M, Kokocinski F, Aken BL, Barrell D, Zadissa A, Searle S, 2012 GENCODE: the reference human genome annotation for the ENCODE Project. Genome Res 22: 1760–1774. 10.1101/gr.135350.111 22955987 PMC3431492

[GR238444LORC26] Hill JT, Demarest BL, Bisgrove BW, Su YC, Smith M, Yost HJ. 2014 Poly Peak Parser: method and software for identification of unknown indels using Sanger sequencing of polymerase chain reaction products. Dev Dyn 243: 1632–1636. 10.1002/dvdy.24183 25160973 PMC4525701

[GR238444LORC27] Houdayer C, Caux-Moncoutier V, Krieger S, Barrois M, Bonnet F, Bourdon V, Bronner M, Buisson M, Coulet F, Gaildrat P, 2012 Guidelines for splicing analysis in molecular diagnosis derived from a set of 327 combined *in silico*/*in vitro* studies on *BRCA1* and *BRCA2* variants. Hum Mutat 33: 1228–1238. 10.1002/humu.22101 22505045

[GR238444LORC28] Huber W, Carey VJ, Gentleman R, Anders S, Carlson M, Carvalho BS, Bravo HC, Davis S, Gatto L, Girke T, 2015 Orchestrating high-throughput genomic analysis with Bioconductor. Nat Methods 12: 115–121. 10.1038/nmeth.3252 25633503 PMC4509590

[GR238444LORC29] Iossifov I, O'Roak BJ, Sanders SJ, Ronemus M, Krumm N, Levy D, Stessman HA, Witherspoon KT, Vives L, Patterson KE, 2014 The contribution of *de novo* coding mutations to autism spectrum disorder. Nature 515: 216–221. 10.1038/nature13908 25363768 PMC4313871

[GR238444LORC30] Ito K, Patel PN, Gorham JM, McDonough B, DePalma SR, Adler EE, Lam L, MacRae CA, Mohiuddin SM, Fatkin D, 2017 Identification of pathogenic gene mutations in *LMNA* and *MYBPC3* that alter RNA splicing. Proc Natl Acad Sci 114: 7689–7694. 10.1073/pnas.1707741114 28679633 PMC5528995

[GR238444LORC31] Jian X, Boerwinkle E, Liu X. 2014a *In silico* prediction of splice-altering single nucleotide variants in the human genome. Nucleic Acids Res 42: 13534–13544. 10.1093/nar/gku1206 25416802 PMC4267638

[GR238444LORC32] Jian X, Boerwinkle E, Liu X. 2014b In silico tools for splicing defect prediction: a survey from the viewpoint of end users. Genet Med 16: 497–503. 10.1038/gim.2013.176 24263461 PMC4029872

[GR238444LORC33] Karolchik D, Hinrichs AS, Furey TS, Roskin KM, Sugnet CW, Haussler D, Kent WJ. 2004 The UCSC Table Browser data retrieval tool. Nucleic Acids Res 32: D493–D496. 10.1093/nar/gkh103 14681465 PMC308837

[GR238444LORC34] Ke S, Shang S, Kalachikov SM, Morozova I, Yu L, Russo JJ, Ju J, Chasin LA. 2011 Quantitative evaluation of all hexamers as exonic splicing elements. Genome Res 21: 1360–1374. 10.1101/gr.119628.110 21659425 PMC3149502

[GR238444LORC35] Ke S, Anquetil V, Zamalloa JR, Maity A, Yang A, Arias MA, Kalachikov S, Russo JJ, Ju J, Chasin LA. 2018 Saturation mutagenesis reveals manifold determinants of exon definition. Genome Res 28: 11–24. 10.1101/gr.219683.116 29242188 PMC5749175

[GR238444LORC36] Kircher M, Witten DM, Jain P, O'Roak BJ, Cooper GM, Shendure J. 2014 A general framework for estimating the relative pathogenicity of human genetic variants. Nat Genet 46: 310–315. 10.1038/ng.2892 24487276 PMC3992975

[GR238444LORC37] Köhler S, Vasilevsky NA, Engelstad M, Foster E, McMurry J, Aymé S, Baynam G, Bello SM, Boerkoel CF, Boycott KM, 2017 The Human Phenotype Ontology in 2017. Nucleic Acids Res 45: D865–D876. 10.1093/nar/gkw1039 27899602 PMC5210535

[GR238444LORC38] Krawczak M, Thomas NS, Hundrieser B, Mort M, Wittig M, Hampe J, Cooper DN. 2007 Single base-pair substitutions in exon–intron junctions of human genes: nature, distribution, and consequences for mRNA splicing. Hum Mutat 28: 150–158. 10.1002/humu.20400 17001642

[GR238444LORC39] Landrum MJ, Lee JM, Benson M, Brown G, Chao C, Chitipiralla S, Gu B, Hart J, Hoffman D, Hoover J, 2016 ClinVar: public archive of interpretations of clinically relevant variants. Nucleic Acids Res 44: D862–D868. 10.1093/nar/gkv1222 26582918 PMC4702865

[GR238444LORC40] Lastella P, Surdo NC, Resta N, Guanti G, Stella A. 2006 In silico and in vivo splicing analysis of *MLH1* and *MSH2* missense mutations shows exon- and tissue-specific effects. BMC Genomics 7: 243 10.1186/1471-2164-7-243 16995940 PMC1590028

[GR238444LORC41] Lek M, Karczewski KJ, Minikel EV, Samocha KE, Banks E, Fennell T, O'Donnell-Luria AH, Ware JS, Hill AJ, Cummings BB, 2016 Analysis of protein-coding genetic variation in 60,706 humans. Nature 536: 285–291. 10.1038/nature19057 27535533 PMC5018207

[GR238444LORC42] Lewandowska MA. 2013 The missing puzzle piece: splicing mutations. Int J Clin Exp Pathol 6: 2675–2682. 24294354 PMC3843248

[GR238444LORC43] Li H, Durbin R. 2009 Fast and accurate short read alignment with Burrows–Wheeler transform. Bioinformatics 25: 1754–1760. 10.1093/bioinformatics/btp324 19451168 PMC2705234

[GR238444LORC44] Liu HX, Cartegni L, Zhang MQ, Krainer AR. 2001 A mechanism for exon skipping caused by nonsense or missense mutations in *BRCA1* and other genes. Nat Genet 27: 55–58. 10.1038/83762 11137998

[GR238444LORC45] Lorson CL, Hahnen E, Androphy EJ, Wirth B. 1999 A single nucleotide in the *SMN* gene regulates splicing and is responsible for spinal muscular atrophy. Proc Natl Acad Sci 96: 6307–6311. 10.1073/pnas.96.11.6307 10339583 PMC26877

[GR238444LORC46] Maslen C, Babcock D, Raghunath M, Steinmann B. 1997 A rare branch-point mutation is associated with missplicing of fibrillin-2 in a large family with congenital contractural arachnodactyly. Am J Hum Genet 60: 1389–1398. 10.1086/515472 9199560 PMC1716103

[GR238444LORC47] McCarthy DJ, Humburg P, Kanapin A, Rivas MA, Gaulton K, Cazier JB, Donnelly P. 2014 Choice of transcripts and software has a large effect on variant annotation. Genome Med 6: 26 10.1186/gm543 24944579 PMC4062061

[GR238444LORC48] McKenna A, Hanna M, Banks E, Sivachenko A, Cibulskis K, Kernytsky A, Garimella K, Altshuler D, Gabriel S, Daly M, 2010 The Genome Analysis Toolkit: a MapReduce framework for analyzing next-generation DNA sequencing data. Genome Res 20: 1297–1303. 10.1101/gr.107524.110 20644199 PMC2928508

[GR238444LORC49] McLaren W, Gil L, Hunt SE, Riat HS, Ritchie GR, Thormann A, Flicek P, Cunningham F. 2016 The Ensembl Variant Effect Predictor. Genome Biol 17: 122 10.1186/s13059-016-0974-4 27268795 PMC4893825

[GR238444LORC50] Mercer TR, Clark MB, Andersen SB, Brunck ME, Haerty W, Crawford J, Taft RJ, Nielsen LK, Dinger ME, Mattick JS. 2015 Genome-wide discovery of human splicing branchpoints. Genome Res 25: 290–303. 10.1101/gr.182899.114 25561518 PMC4315302

[GR238444LORC51] R Core Team. 2018 R: a language and environment for statistical computing. R Foundation for Statistical Computing, Vienna https://www.R-project.org/.

[GR238444LORC52] Ramu A, Noordam MJ, Schwartz RS, Wuster A, Hurles ME, Cartwright RA, Conrad DF. 2013 DeNovoGear: *de novo* indel and point mutation discovery and phasing. Nat Methods 10: 985–987. 10.1038/nmeth.2611 23975140 PMC4003501

[GR238444LORC53] Samocha KE, Robinson EB, Sanders SJ, Stevens C, Sabo A, McGrath LM, Kosmicki JA, Rehnstrom K, Mallick S, Kirby A, 2014 A framework for the interpretation of *de novo* mutation in human disease. Nat Genet 46: 944–950. 10.1038/ng.3050 25086666 PMC4222185

[GR238444LORC54] Sangermano R, Khan M, Cornelis SS, Richelle V, Albert S, Elmelik D, Garanto A, Qamar R, Lugtenberg D, van den Born LI, 2018 *ABCA4* midigenes reveal the full splice spectrum of all reported noncanonical splice site variants in Stargardt disease. Genome Res 28: 100–110. 10.1101/gr.226621.117 29162642 PMC5749174

[GR238444LORC55] Scotti MM, Swanson MS. 2016 RNA mis-splicing in disease. Nat Rev Genet 17: 19–32. 10.1038/nrg.2015.3 26593421 PMC5993438

[GR238444LORC56] Short PJ, McRae JF, Gallone G, Sifrim A, Won H, Geschwind DH, Wright CF, Firth HV, FitzPatrick DR, Barrett JC, 2018 *De novo* mutations in regulatory elements in neurodevelopmental disorders. Nature 555: 611–616. 10.1038/nature25983 29562236 PMC5912909

[GR238444LORC57] Singh T, Kurki MI, Curtis D, Purcell SM, Crooks L, McRae J, Suvisaari J, Chheda H, Blackwood D, Breen G, 2016 Rare loss-of-function variants in *SETD1A* are associated with schizophrenia and developmental disorders. Nat Neurosci 19: 571–577. 10.1038/nn.4267 26974950 PMC6689268

[GR238444LORC58] Soemedi R, Cygan KJ, Rhine CL, Wang J, Bulacan C, Yang J, Bayrak-Toydemir P, McDonald J, Fairbrother WG. 2017 Pathogenic variants that alter protein code often disrupt splicing. Nat Genet 49: 848–855. 10.1038/ng.3837 28416821 PMC6679692

[GR238444LORC59] Soukarieh O, Gaildrat P, Hamieh M, Drouet A, Baert-Desurmont S, Frébourg T, Tosi M, Martins A. 2016 Exonic splicing mutations are more prevalent than currently estimated and can be predicted by using *in silico* tools. PLoS Genet 12: e1005756 10.1371/journal.pgen.1005756 26761715 PMC4711968

[GR238444LORC60] Taggart AJ, Lin CL, Shrestha B, Heintzelman C, Kim S, Fairbrother WG. 2017 Large-scale analysis of branchpoint usage across species and cell lines. Genome Res 27: 639–649. 10.1101/gr.202820.115 28119336 PMC5378181

[GR238444LORC61] Tang R, Prosser DO, Love DR. 2016 Evaluation of bioinformatic programmes for the analysis of variants within splice site consensus regions. Adv Bioinformatics 2016: 5614058 10.1155/2016/5614058 27313609 PMC4894998

[GR238444LORC62] Teraoka SN, Telatar M, Becker-Catania S, Liang T, Onengut S, Tolun A, Chessa L, Sanal O, Bernatowska E, Gatti RA, 1999 Splicing defects in the ataxia-telangiectasia gene, *ATM:* underlying mutations and consequences. Am J Hum Genet 64: 1617–1631. 10.1086/302418 10330348 PMC1377904

[GR238444LORC63] Thery JC, Krieger S, Gaildrat P, Revillion F, Buisine MP, Killian A, Duponchel C, Rousselin A, Vaur D, Peyrat JP, 2011 Contribution of bioinformatics predictions and functional splicing assays to the interpretation of unclassified variants of the *BRCA* genes. Eur J Hum Genet 19: 1052–1058. 10.1038/ejhg.2011.100 21673748 PMC3190263

[GR238444LORC64] van der Klift HM, Jansen AM, van der Steenstraten N, Bik EC, Tops CM, Devilee P, Wijnen JT. 2015 Splicing analysis for exonic and intronic mismatch repair gene variants associated with Lynch syndrome confirms high concordance between minigene assays and patient RNA analyses. Mol Genet Genomic Med 3: 327–345. 10.1002/mgg3.145 26247049 PMC4521968

[GR238444LORC65] Vaz-Drago R, Custodio N, Carmo-Fonseca M. 2017 Deep intronic mutations and human disease. Hum Genet 136: 1093–1111. 10.1007/s00439-017-1809-4 28497172

[GR238444LORC66] Wang Y, Wang Z. 2014 Systematical identification of splicing regulatory *cis*-elements and cognate *trans*-factors. Methods 65: 350–358. 10.1016/j.ymeth.2013.08.019 23974071 PMC3932149

[GR238444LORC67] Wang K, Li M, Hakonarson H. 2010 ANNOVAR: functional annotation of genetic variants from high-throughput sequencing data. Nucleic Acids Res 38: e164 10.1093/nar/gkq603 20601685 PMC2938201

[GR238444LORC68] Wright CF, Fitzgerald TW, Jones WD, Clayton S, McRae JF, van Kogelenberg M, King DA, Ambridge K, Barrett DM, Bayzetinova T, 2015 Genetic diagnosis of developmental disorders in the DDD study: a scalable analysis of genome-wide research data. Lancet 385: 1305–1314. 10.1016/S0140-6736(14)61705-0 25529582 PMC4392068

[GR238444LORC69] Xiong HY, Alipanahi B, Lee LJ, Bretschneider H, Merico D, Yuen RK, Hua Y, Gueroussov S, Najafabadi HS, Hughes TR, 2015 RNA splicing. The human splicing code reveals new insights into the genetic determinants of disease. Science 347: 1254806 10.1126/science.1254806 25525159 PMC4362528

[GR238444LORC70] Yeo G, Burge CB. 2004 Maximum entropy modeling of short sequence motifs with applications to RNA splicing signals. J Comput Biol 11: 377–394. 10.1089/1066527041410418 15285897

[GR238444LORC71] Zhang XH, Chasin LA. 2004 Computational definition of sequence motifs governing constitutive exon splicing. Genes Dev 18: 1241–1250. 10.1101/gad.1195304 15145827 PMC420350

