# Supplementary material for: Pathogenicity and selective constraint on variation near splice sites
Source: Genome Res. 2019 Feb;29(2):159–70. doi: 10.1101/gr.238444.118 (PMC6360807; doi:10.1101/gr.238444.118)
Supplement: Supplemental Material [file supp_29_2_159__index.html]

Pathogenicity and selective constraint on variation near splice sites — Pathogenicity and selective constraint on variation near splice sites — Supplemental Material 

# Pathogenicity and selective constraint on variation near splice sites

## Supplemental Material

- Supplemental\_information\_and\_figures.pdf
- Supplemental\_Table\_S4.pdf
- Supplemental\_code\_and\_data.zip
