# Supplementary material for: Pathogenicity and selective constraint on variation near splice sites
Source: Genome Res. 2019 Feb;29(2):159–70. doi: 10.1101/gr.238444.118 (PMC6360807; doi:10.1101/gr.238444.118)
Supplement: Supplemental Material [file supp_gr.238444.118_Supplemental_information_and_figures.pdf]

Supplemental Material – Table of contents

Supplemental Fig. S1 - Patterns of selection in the polypyrimidine tract (page 2-3)

Supplemental Fig. S2 – Enrichment of *de novo* mutations in diagnosed and undiagnosed probands (page 4)

Supplemental Fig. S3 – Relationship between positive predictive values and population genetics metrics (page 5-6)

Supplemental Table S1 - *De novo* mutations in non-canonical near splice positions not thought to be diagnostic (page 7)

Supplemental Table S2 - Phenotypic data on probands with likely diagnostic near-splice *de novo* mutations (page 8-9)

Supplemental Table S3 – Splicing pathogenicity scores for near-splice *de novo* SNVs (page 10-11)

Supplemental Fig. S4 – Outcomes of minigene assays for splicing validations (page 12-15)

Supplemental Fig. S5 – Proportion of parental variants in high pLI gene by pathogenicity score (page 16-17)

Supplemental Fig. S6 – Mutability adjusted proportion of singletons by pathogenicity score, excluding canonical splice sites (page 18-19)

Supplemental Fig. S7 – Proportion of parental variants in high pLI gene by pathogenicity score, excluding canonical splice sites (page 20-21)

Supplemental Table S5 – Details of variants selected for validation assay (page 22)

Supplemental Table S6 – Primers used to amplify region of interest from patient DNA (page 23)

Supplemental Table S7 - Primers to PCR amplify ref and alt intron with Gibson overhangs (page 24)

Supplemental Table S8 – PCR primers to amplify vector backbone (page 25)

Supplemental Table S9 - RT-PCR primers (page 25)

Supplemental Table S10 - Sequencing primers (page 25)

NB – due to its size, Supplemental Table S4 is provided as a separate Excel file

Supplemental Fig. S1 – Patterns of selection in the polypyrimidine tract

Mutability adjusted proportion of singletons (MAPS), with 95% confidence intervals (CI) across region upstream of the acceptor site (including polypyrimidine tract region) split by changes from a pyrimidine to a purine (PyPu) vs all other changes in DDD unaffected parents (A) and ExAC data (B). Deficit of variants in genes with high pLI in unaffected parents recruited as part of DDD study across region upstream of the acceptor site (including polypyrimidine tract region) split by changes from a pyrimidine to a purine (PyPu) vs all other changes (C).

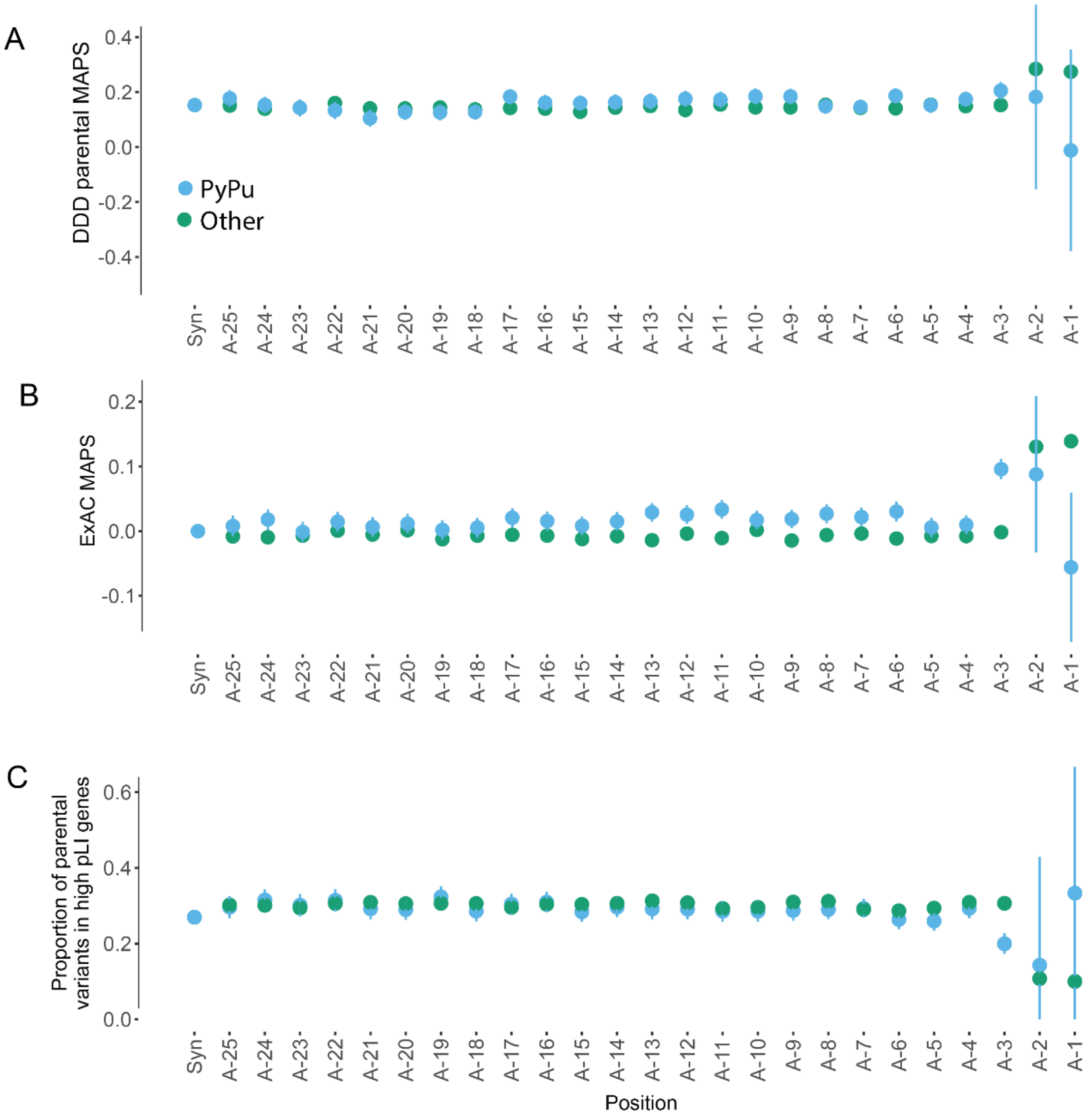

A
DDD parental MAPS
0.4
0.2
0.0
-0.2
-0.4
PyPu
Other
Syn
A-25
A-24
A-23
A-22
A-21
A-20
A-19
A-18
A-17
A-16
A-15
A-14
A-13
A-12
A-11
A-10
A-9
A-8
A-7
A-6
A-5
A-4
A-3
A-2
A-1
B
ExAC MAPS
0.2
0.1
0.0
-0.1
C
Proportion of parental variants in high pLI genes
0.6
0.4
0.2
0.0
Position

Supplemental Fig. S2 – Enrichment of *de novo* mutations in diagnosed and undiagnosed probands

Enrichment of *de novo* mutations (DNMs) in dominant DD-associated genes across the splicing region in 1417 DDD probands with a clinician confirmed diagnosis (Diagnosed cases) and 3364 DDD probands lacking a potentially diagnostic variant (Undiagnosed cases), with FDR corrected Poisson p-values. Splice acceptor and splice donor consensus sequences shown below, as in Figure 1.

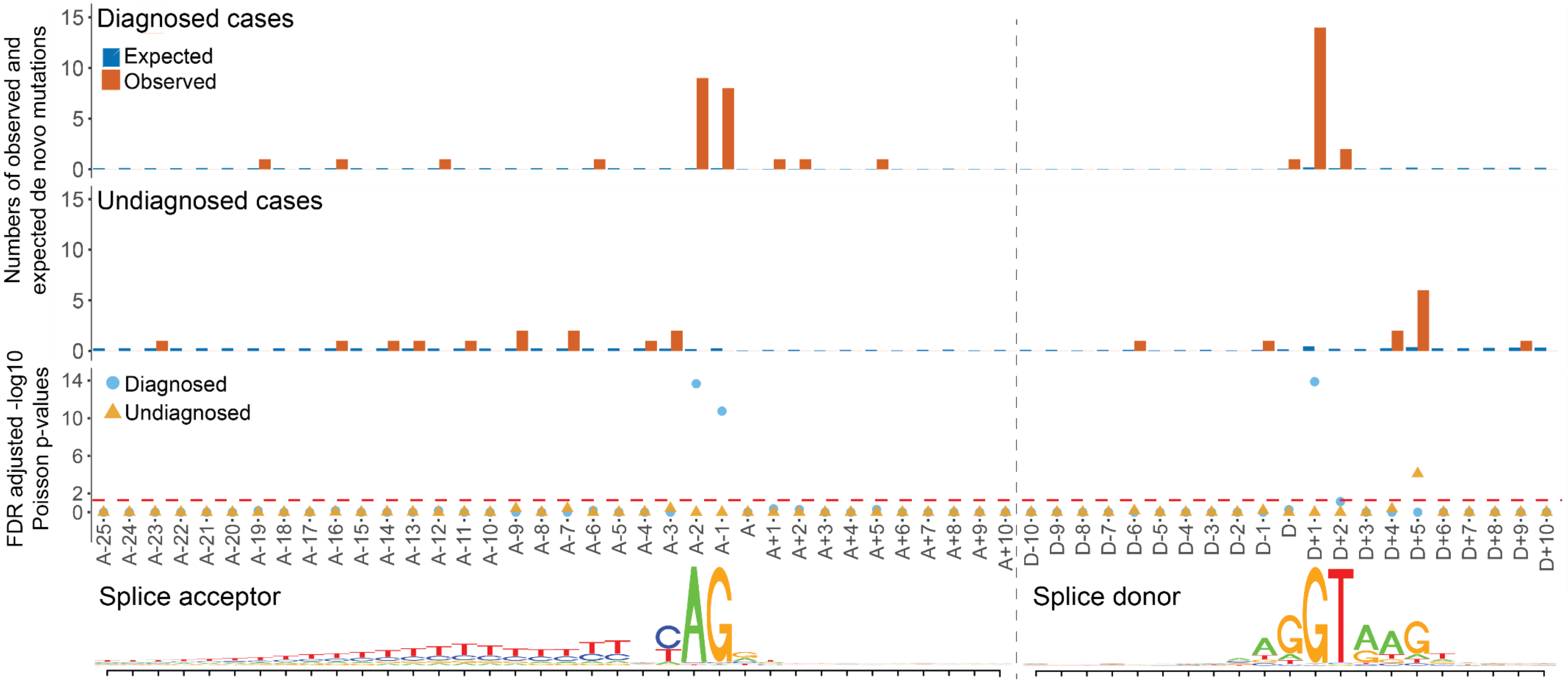

Supplemental Fig. S3 – Relationship between positive predictive values and population genetics metrics

Relationship between calculated positive predictive values (PPVs) for classes of splice and non-splice mutations, based on enrichment of DNMs, with population based metrics MAPS and 95% CI (in DDD unaffected parents and ExAC data) and proportion (with 95% CI) of unaffected DDD parental variants in high pLI genes (pLI > 0.9)

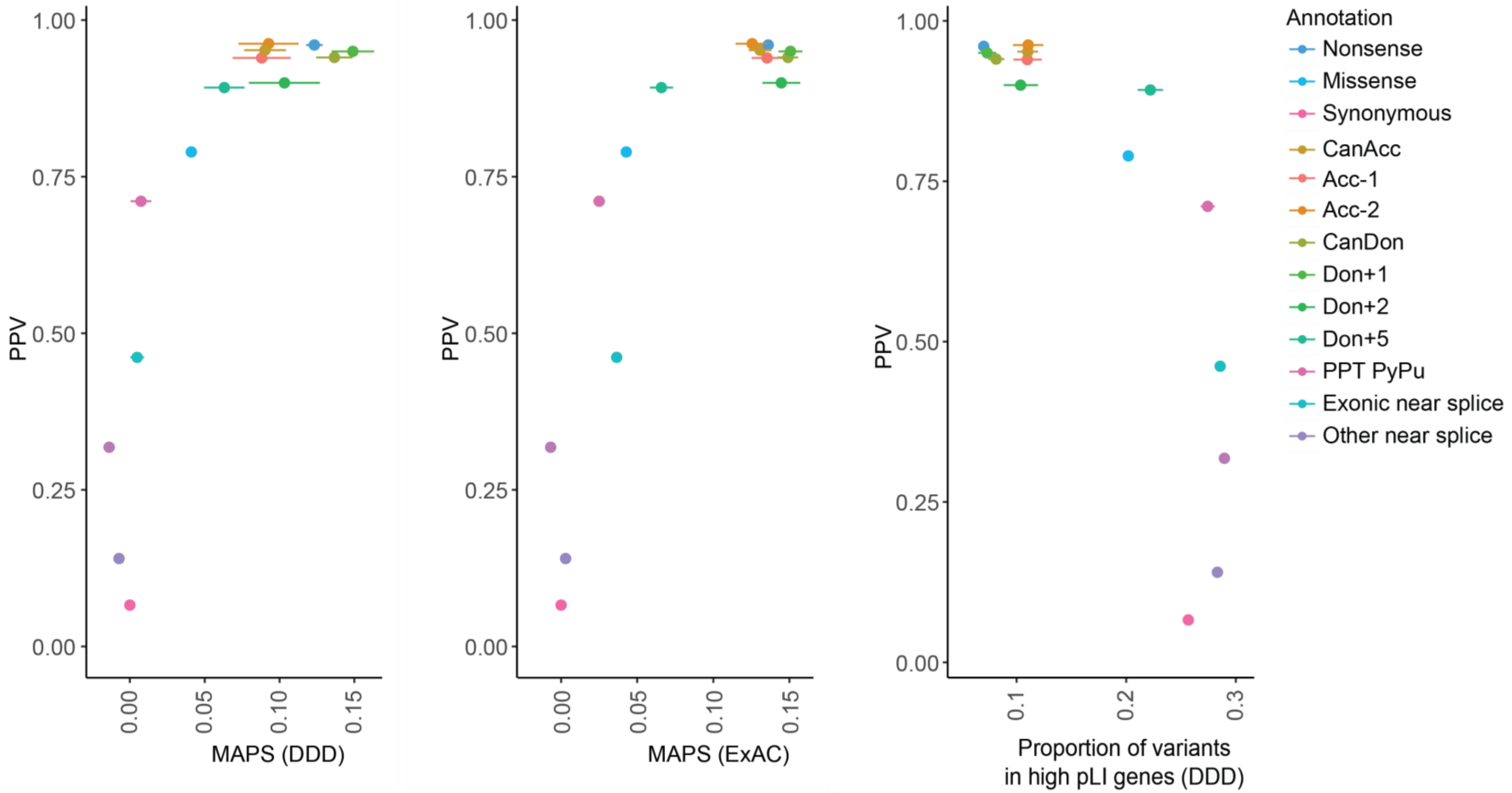
PPV
1.00
0.75
0.50
0.25
0.00
0.00
0.05
0.10
0.15
MAPS (DDD)
MAPS (ExAC)
0.1
0.2
0.3
Proportion of variants
in high pLI genes (DDD)
Annotation
Nonsense
Missense
Synonymous
CanAcc
Acc-1
Acc-2
CanDon
Don+1
Don+2
Don+5
PPT PyPu
Exonic near splice
Other near splice

Supplemental Table S1 - *De novo* mutations in non-canonical near splice positions not thought to be diagnostic

Genomic coordinates and annotations of 20 *de novo* splice region variants identified in undiagnosed DDD probands in known dominant DD-associated genes, deemed unlikely to be diagnostic based on lack of phenotypic match between proband and associated syndrome (hg19 coordinates)

Variant	Gene	VEP annotation	Splice Annotation	Clinical classification
18:42618432_G/T	*SETBP1*	intron_variant	acc-18	Likely benign
3:38988415_AC/A	*SCN11A*	intron_variant	acc-17	Likely benign
17:38240072_A/G	*THRA*	intron_variant	acc-16	Likely benign
19:13387958_G/A	*CACNA1A*	intron_variant	acc-16	Likely benign
3:189456422_A/G	*TP63*	intron_variant	acc-9	Likely benign
1:7309543_GTTT/GTT	*CAMTA1*	splice_region_variant	acc-8	Likely benign
16:30745810_C/G	*SRCAP*	splice_region_variant	acc-7	Likely benign
16:29816431_G/A	*KIF22*	splice_region_variant	acc-5	Likely benign
22:41543944_C/T	*EP300*	synonymous_variant	don-6	Likely benign
10:94381235_G/T	*KIF11*	splice_region_variant	don+5	Likely benign
16:2129206_G/A	*TSC2*	intron_variant	don+9	Likely benign
2:158594942_G/T	*ACVR1*	intron_variant	don+10	Likely benign
19:50912018_C/T	*POLD1*	synonymous_variant	acc-24	Uncertain
3:41266439_T/G	*CTNNB1*	splice_region_variant	acc-6	Uncertain
17:44159911_GC/G	*KANSL1*	splice_region_variant	acc-3	Uncertain
3:71021701_C/T	*FOXP1*	splice_region_variant	don+5	Uncertain
6:157431700_G/A	*ARID1B*	splice_region_variant	don+5	Uncertain
X:41196724_T/G	*DDX3X*	splice_region_variant	don+6	Uncertain
3:111366523_A/C	*CD96*	intron_variant	don+10	Uncertain
8:117869033_G/C	*RAD21*	intron_variant	acc-23	Uncertain

Supplemental Table S2 - Phenotypic data on probands with likely diagnostic near-splice *de novo* mutations (DNMs) (hg19 coordinates)

*HPO = Human Phenotype Ontology

**chrom:pos_ref/alt**	**symbol**	**Splice annotation**	**HPO* terms**	**HPO* terms (translation)**
7:42063221_G/C	*GLI3*	Acc-14	HP:0001841, HP:0010709, HP:0011304	2-4 finger syndactyly, Broad thumb, Preaxial foot polydactyly
16:3819367_C/T	*CREBBP*	Acc-13	HP:0000028, HP:0000179, HP:0000248, HP:0000252, HP:0000347, HP:0000486, HP:0001263, HP:0001510, HP:0001831, HP:0002019, HP:0002205, HP:0004691, HP:0011304	2-3 toe syndactyly, Brachycephaly, Broad thumbs, Constipation, Cryptorchidism, Global developmental delay, Growth delay, Microcephaly, Micrognathia, Recurrent respiratory infections, Short toes, Strabismus, Thick lower lip vermilion
22:24143120_T/G	*SMARCB1*	Acc-11	HP:0000294, HP:0000680, HP:0000696, HP:0000750, HP:0001263, HP:0001763, HP:0001999, HP:0002205, HP:0002213, HP:0007021, HP:0007096, HP:0010830, HP:0010877, HP:0100543, HP:0003045, HP:0002926, HP:0000708, HP:0000545, HP:0002164, HP:0001212	Unilateral strabismus, Delayed eruption of permanent teeth, Delayed eruption of primary teeth, Delayed speech and language development, Global developmental delay, Cognitive impairment, Impaired tactile sensation, Pain insensitivity, Pes planus, Recurrent respiratory infections, Fine hair, Low anterior hairline, Abnormal facial shape, Hypoplasia of the optic tract, Abnormality of the patella, Abnormality of thyroid physiology, Behavioural abnormality, Myopia , Nail dysplasia, Prominent fingertip pads
18:52895603_T/C	*TCF4*	Acc-11	HP:0000122, HP:0000252, HP:0000545, HP:0000646, HP:0001263, HP:0001999	Abnormal facial shape, Amblyopia, Global developmental delay, Microcephaly, Myopia, Unilateral renal agenesis
5:88025173_A/C	*MEF2C*	Acc-9	HP:0000179, HP:0001250, HP:0002500, HP:0006579, HP:0011344, HP:0100023	Abnormality of the cerebral white matter, Prolonged neonatal jaundice, Recurrent hand flapping, Seizures, Severe global developmental delay, Thick lower lip vermilion
9:130988306_G/A	*DNM1*	Acc-8	HP:0001250, HP:0001263, HP:0001319, HP:0009117, HP:0011228, HP:0011344, HP:0011947	Aplasia/Hypoplasia of the maxilla, Global developmental delay, Horizontal eyebrow, Neonatal hypotonia, Respiratory tract infection, Seizures, Severe global developmental delay
8:61763045_G/A	*CHD7*	Acc-7	HP:0000185, HP:0000202, HP:0000589, HP:0001263, HP:0002564, HP:0003508, HP:0011678	Cleft soft palate, Coloboma, Global developmental delay, Oral cleft, Proportionate short stature, Tetralogy of Fallot with pulmonary atresia and major aortopulmonary collateral arteries, obsolete Malformation of the heart and great vessels
17:38801875_T/C	*SMARCE1*	Acc-4	HP:0000750, HP:0004322, HP:0005484	Delayed speech and language development, Postnatal microcephaly, Short stature
1:27097607_C/A	*ARID1A*	Acc-3	HP:0000179, HP:0000347, HP:0000364, HP:0000369, HP:0000377, HP:0000490, HP:0000973, HP:0001338, HP:0001511, HP:0008935	Abnormality of the pinna, Cutis laxa, Deeply set eye, Generalized neonatal hypotonia, Hearing abnormality, Intrauterine growth retardation, Low-set ears, Micrognathia, Partial agenesis of the corpus callosum, Thick lower lip vermilion
9:140728798_C/G	*EHMT1*	Acc-3	HP:0002558, HP:0002020, HP:0002021, HP:0012716, HP:0008915, HP:0000248, HP:0030812, HP:0001800, HP:0012433, HP:0100543, HP:0000750, HP:0040082, HP:0010864, HP:0005100	Supernumerary nipple, Gastroesophageal reflux, Pyloric stenosis, Moderate conductive hearing impairment, Truncal obesity, Brachycephaly, Enlarged tonsils, Hypoplastic toenails, Abnormal social behaviour, Cognitive impairment, Delayed speech and language development, Happy demeanor, Intellectual disability, severe, Premature birth following premature rupture of fetal membranes

2:223160248_T/C	*PAX3*	Don-1	HP:0000218, HP:0000316, HP:0000460, HP:0000527, HP:0000581, HP:0000582, HP:0002829, HP:0007429, HP:0007603, HP:0009889, HP:0000426, HP:0008573, HP:0000579, HP:0000402	Arthralgia, Blepharophimosis, Few cafe-au-lait spots, Freckles in sun-exposed areas, High palate, Hypertelorism, Localized hirsutism, Long eyelashes, Narrow nose, Upslanted palpebral fissure, High nasal bridge, Low-frequency sensorineural hearing impairment, Lacrimal duct obstruction, Narrow ear canal
2:166229861_A/G	*SCN2A*	Don+4	HP:0000717, HP:0001344, HP:0002342, HP:0001250	Absent speech, Autism, Intellectual disability, moderate, Seizures
9:130422391_A/G	*STXBP1*	Don+4	HP:0001048, HP:0001252, HP:0002599, HP:0011344	Cavernous hemangioma, Head titubation, Muscular hypotonia, Severe global developmental delay
22:41556731_G/A	*EP300*	Don+5	HP:0000023, HP:0000213, HP:0000220, HP:0000322, HP:0000369, HP:0000414, HP:0000486, HP:0000490, HP:0000527, HP:0001263, HP:0001537, HP:0001771, HP:0005484, HP:0007993, HP:0008551, HP:0008850, HP:0100023, HP:0000717	Achilles tendon contracture, Bulbous nose, Deeply set eye, Global developmental delay, Inguinal hernia, Long eyelashes, Low-set ears, Malformed lacrimal ducts, Microtia, Postnatal microcephaly, Recurrent hand flapping, Severe postnatal growth retardation, Short philtrum, Strabismus, Thin vermilion border, Umbilical hernia, Velopharyngeal insufficiency, Autism
2:149221493_G/C	*MBD5*	Don+5	HP:0000252, HP:0000664, HP:0001601, HP:0002020	Gastroesophageal reflux, Laryngomalacia, Microcephaly, Synophrys
9:130427615_G/C	*STXBP1*	Don+5	HP:0000733, HP:0002066, HP:0002378, HP:0002943, HP:0003763, HP:0007359, HP:0010864	Bruxism, Focal seizures, Gait ataxia, Hand tremor, Intellectual disability, severe, Stereotypy, Thoracic scoliosis
17:42956919_C/T	*EFTUD2*	Don+5	HP:0000253, HP:0000286, HP:0000384, HP:0000396, HP:0000412, HP:0011343	Epicanthus, Moderate global developmental delay, Overfolded helix, Preauricular skin tag, Progressive microcephaly, Protruding ear
20:61452890_C/G	*COL9A3*	Don+8	HP:0000729, HP:0000750, HP:0010529, HP:0000735, HP:0001382, HP:0008947, HP:0000736, HP:0000733	Autistic behavior, Delayed speech and language development, Echolalia, Impaired social interactions, Joint hypermobility, Muscular hypotonia, Short attention span, Stereotypy

Supplemental Table S3 – Splicing pathogenicity scores for near-splice *de novo* SNVs (hg19 coordinates)

chrom:pos_ref/alt	Symbol	Splice Annotation	AdaBoost	Random Forest	MES (Percent difference)	Spidex (dpsi_max_tissue)	CADD	Likely diagnostic
7:42063221_G/C	*GLI3*	acc-14	NA	NA	-24.4305	0.2226	0.2030	Yes
16:3819367_C/T	*CREBBP*	acc-13	NA	NA	-45.4327	NA	7.8700	Yes
22:24143120_T/G	*SMARCB1*	acc-11	0.0975	0.3360	-20.8763	-6.2151	7.3290	Yes
18:52895603_T/C	*TCF4*	acc-11	0.9979	0.8400	-44.9511	-0.6026	7.7960	Yes
5:88025173_A/C	*MEF2C*	acc-9	0.9999	0.9560	-110.2334	-0.2806	11.1000	Yes
9:130988306_G/A	*DNM1*	acc-8	0.0195	0.0340	-80.8843	-1.1322	19.1500	Yes
8:61763045_G/A	*CHD7*	acc-7	0.9358	NA	-130.4054	0.1808	12.9000	Yes
17:38801875_T/C	*SMARCE1*	acc-4	0.9770	0.6580	-29.4053	-1.0875	8.6300	Yes
1:27097607_C/A	*ARID1A*	acc-3	0.9100	0.8040	-31.3699	NA	14.4500	Yes
9:140728798_C/G	*EHMT1*	acc-3	0.9998	0.9640	-123.7713	-14.8679	5.4730	Yes
2:223160248_T/C	*PAX3*	don-1	0.9999	1.0000	-47.5131	-2.7475	19.7300	Yes
2:166229861_A/G	*SCN2A*	don+4	0.9679	0.7120	-29.9674	-4.8629	15.2500	Yes
9:130422391_A/G	*STXBP1*	don+4	0.9997	0.9700	-75.3149	-6.3753	16.8100	Yes
9:130427615_G/C	*STXBP1*	don+5	0.9980	0.8900	-20.8484	-2.0404	15.3600	Yes
17:42956919_C/T	*EFTUD2*	don+5	1.0000	0.9540	-90.4161	-0.8542	17.6400	Yes
22:41556731_G/A	*EP300*	don+5	0.9997	0.9720	-95.9032	-18.9583	15.7000	Yes
2:149221493_G/C	*MBD5*	don+5	1.0000	0.9980	-78.8978	-1.3005	13.6300	Yes
20:61452890_C/G	*COL9A3*	don+8	0.0004	0.0140	NA	-2.7056	5.6440	Yes
19:50912018_C/T	*POLD1*	acc-24	NA	NA	NA	-0.1984	5.0910	No
8:117869033_G/C	*RAD21*	acc-23	NA	NA	NA	-0.6527	3.1330	No
18:42618432_G/T	*SETBP1*	acc-18	NA	NA	11.7216	NA	4.8110	No
17:38240072_A/G	*THRA*	acc-16	NA	NA	16.0602	0.1011	8.4540	No
19:13387958_G/A	*CACNA1A*	acc-16	NA	NA	-1.6273	-0.3749	3.1510	No
3:189456422_A/G	*TP63*	acc-9	0.0000	0.0040	18.4615	-0.0061	0.9540	No
16:30745810_C/G	*SRCAP*	acc-7	0.0768	0.4380	-29.3853	-1.4174	10.1000	No
3:41266439_T/G	*CTNNB1*	acc-6	0.9817	0.6700	-58.3691	0.5724	7.3830	No

16:29816431_G/A	*KIF22*	acc-5	0.0008	0.0300	-2.5510	-0.9766	11.8200	No
22:41543944_C/T	*EP300*	don-6	NA	NA	NA	-0.8986	13.8500	No
3:71021701_C/T	*FOXP1*	don+5	0.0648	0.1140	-82.0779	-3.0711	16.7400	No
10:94381235_G/T	*KIF11*	don+5	0.7600	0.6240	-33.5208	-0.1237	9.7980	No
6:157431700_G/A	*ARID1B*	don+5	0.9999	0.9840	-72.7838	-13.1815	13.2600	No
X:41196724_T/G	*DDX3X*	don+6	0.9727	0.8920	-48.2911	NA	14.3800	No
16:2129206_G/A	*TSC2*	don+9	NA	NA	NA	1.6113	2.2320	No
2:158594942_G/T	*ACVR1*	don+10	NA	NA	NA	-0.0722	3.6330	No
3:111366523_A/C	*CD96*	don+10	NA	NA	NA	0.1223	9.3260	No

Supplemental Fig. S4 – Outcomes of minigene assays for splicing validations.

A – schematic diagram of splicing construct for PolyPy variants, plus experimental outcome for five PolyPy variants that generated altered splicing products (all generated a cryptic splice site upstream of the CSS, causing retention of part of the intron (in four instances leading to a frameshift effect, and in one leading to the inclusion of two additional amino acids in the protein sequence). B – schematic diagram of splicing construct for don+5 mutation, plus experimental outcome for don+5 mutation, which caused the utilisation of a second "GT" site within the reference sequence as a splice donor site, causing retention of intronic sequence and leading to a frameshift effect. C – validation outcomes for PolyPy DNMs that were not found to affect splicing. D – validation outcomes for parental variants selected as negative controls. NB - For the *CHD7* variant, two splice products were observed, corresponding to the expected (wild type) splicing and the retention of 5bp intronic sequence. The *MBD5* variant gave multiple splice isoforms, with retention of 12bp intronic sequence being the most prevalent, but normally spliced, 19bp intronic retention, and complete intron retention also observed. This figure shows the predominant isoform observed for these variants.

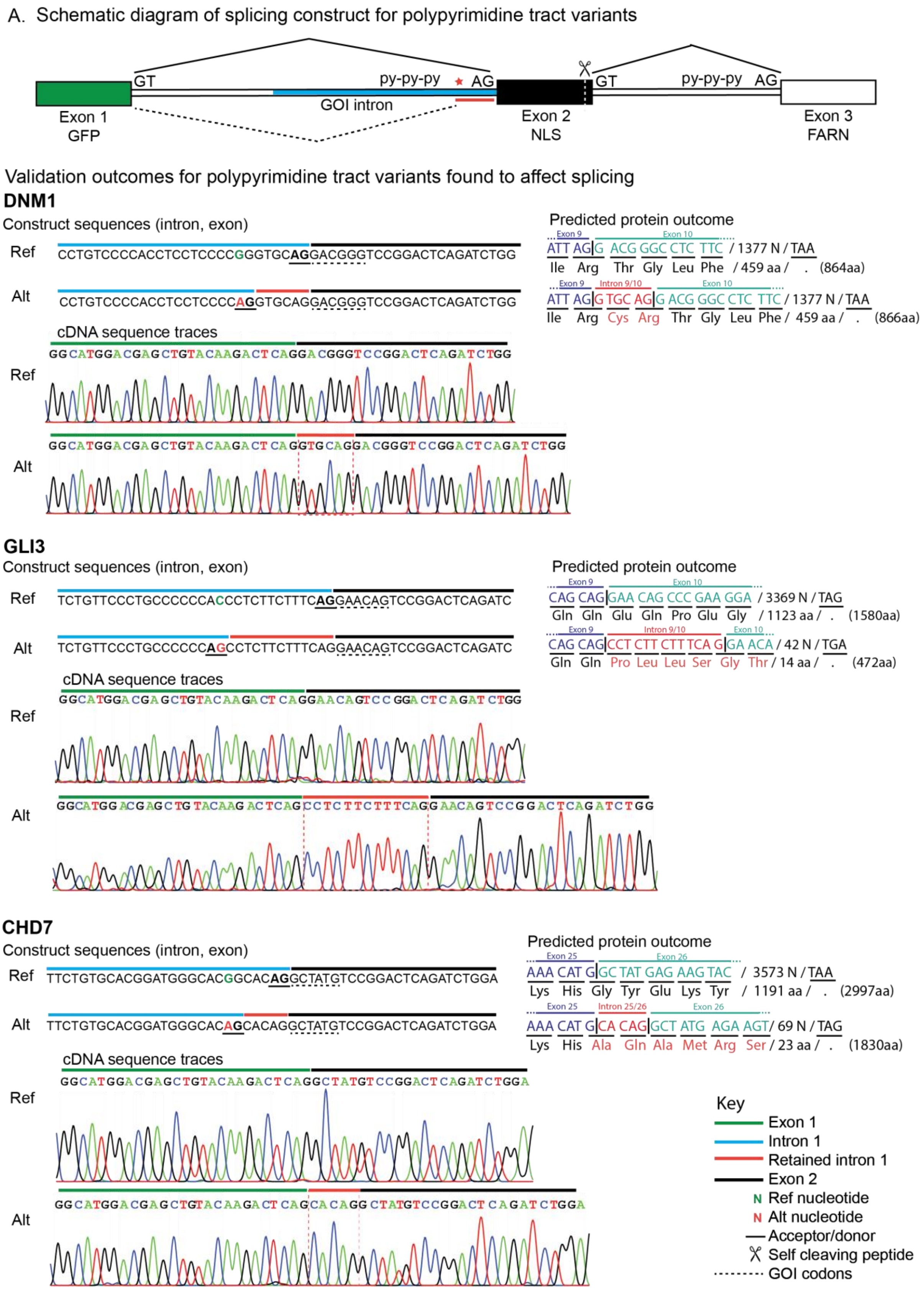
A. Schematic diagram of splicing construct for polypyrimidine tract variants
GT
py-py-py
AG
GOI intron
Exon 1
GFP
Exon 2
NLS
GT
py-py-py
AG
Exon 3
FARN
Validation outcomes for polypyrimidine tract variants found to affect splicing
DNM1
Construct sequences (intron, exon)
Ref
CCTGTCCCCACCTCCTCCCCGGGTGCAGGACGGGTCCGGACTCAGATCTGG
Alt
CCTGTCCCCACCTCCTCCCCAGGTGCAGGACGGGTCCGGACTCAGATCTGG
cDNA sequence traces
Predicted protein outcome
Exon 9
Exon 10
ATT AG|G ACG GGC CTC TTC / 1377 N / TAA
Ile Arg Thr Gly Leu Phe / 459 aa / . (864aa)
Exon 9
Intron 9/10
Exon 10
ATT AG|G TGC AG|G ACG GGC CTC TTC / 1377 N / TAA
Ile Arg Cys Arg Thr Gly Leu Phe / 459 aa / . (866aa)
GLI3
Construct sequences (intron, exon)
Ref
TCTGTTCCCTGCCCCCCACCCTCTTCTTTCAGGAACAGTCCGGACTCAGATC
Alt
TCTGTTCCCTGCCCCCCAGCCTCTTCTTTCAGGAACAGTCCGGACTCAGATC
cDNA sequence traces
Predicted protein outcome
Exon 9
Exon 10
CAG CAG|GAA CAG CCC GAA GGA / 3369 N / TAG
Gln Gln Glu Gln Pro Glu Gly / 1123 aa / . (1580aa)
Exon 9
Intron 9/10
Exon 10
CAG CAG|CCT CTT CTT TCA G|GA ACA / 42 N / TGA
Gln Gln Pro Leu Leu Ser Gly Thr / 14 aa / . (472aa)
CHD7
Construct sequences (intron, exon)
Ref
TTCTGTGCACGGATGGGCACGGCACAGGCTATGTCCGGACTCAGATCTGGA
Alt
TTCTGTGCACGGATGGGCACAGCACAGGCTATGTCCGGACTCAGATCTGGA
cDNA sequence traces
Predicted protein outcome
Exon 25
Exon 26
AAA CAT G|GC TAT GAG AAG TAC / 3573 N / TAA
Lys His Gly Tyr Glu Lys Tyr / 1191 aa / . (2997aa)
Exon 25
Intron 25/26
Exon 26
AAA CAT G|CA CAG|GCT ATG AGA AGT / 69 N / TAG
Lys His Ala Gln Ala Met Arg Ser / 23 aa / . (1830aa)
Key
Exon 1
Intron 1
Retained intron 1
Exon 2
N Ref nucleotide
N Alt nucleotide
Acceptor/donor
Self cleaving peptide
GOI codons

Validation outcomes for polypyrimidine tract variants found to affect splicing (continued)

CREBBP

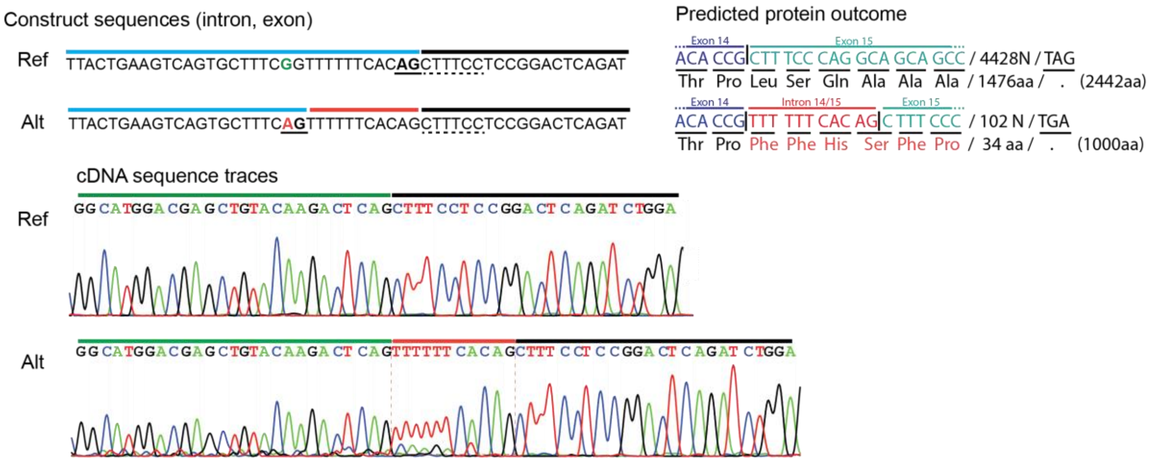

MEF2C

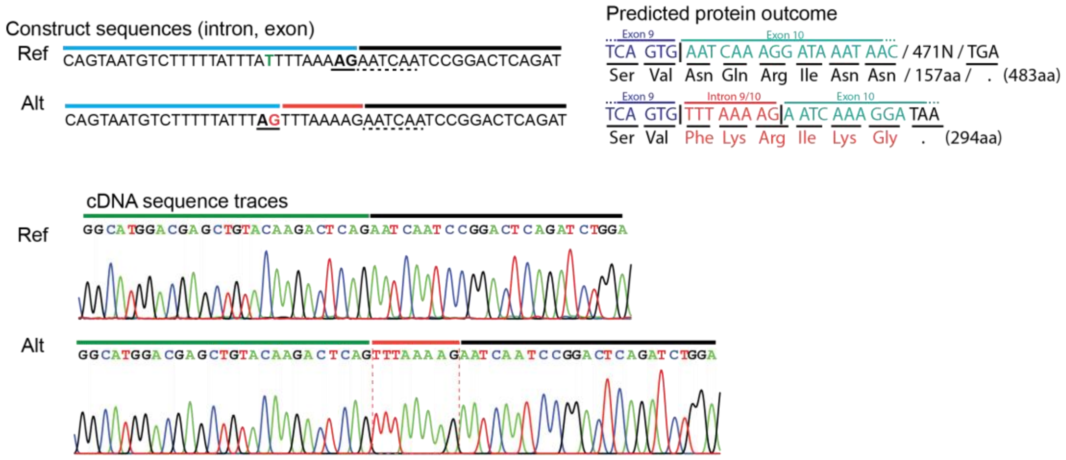

B. Schematic diagram of splicing construct for don+5 variant

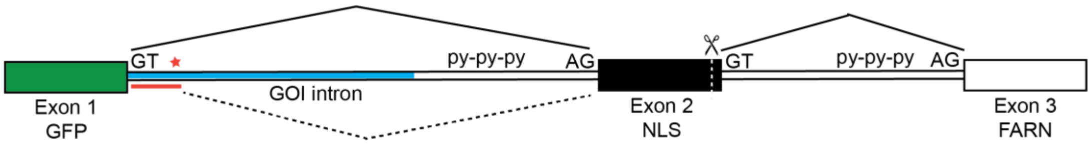

Validation outcomes for don+5 variant found to affect splicing

MBD5

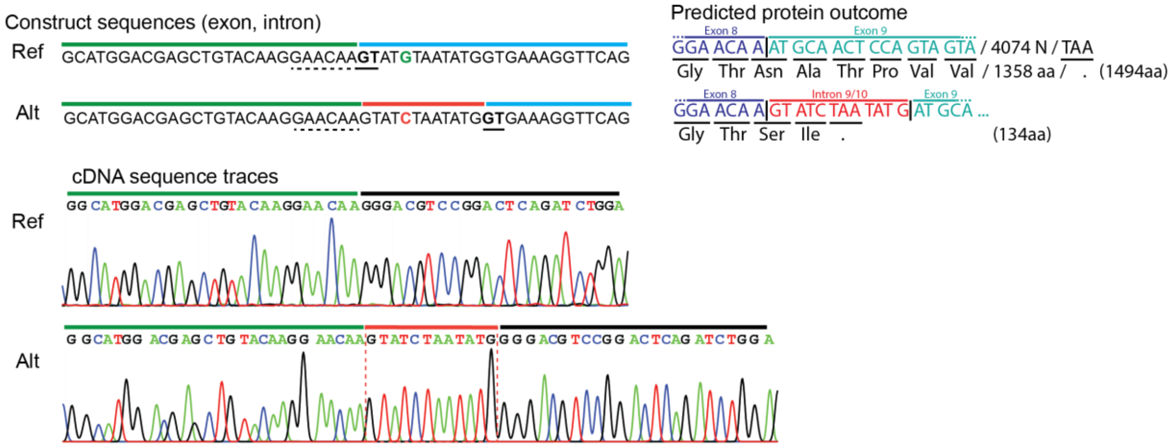

C. Validation outcomes for polypyrimidine tract variants that did not affect splicing

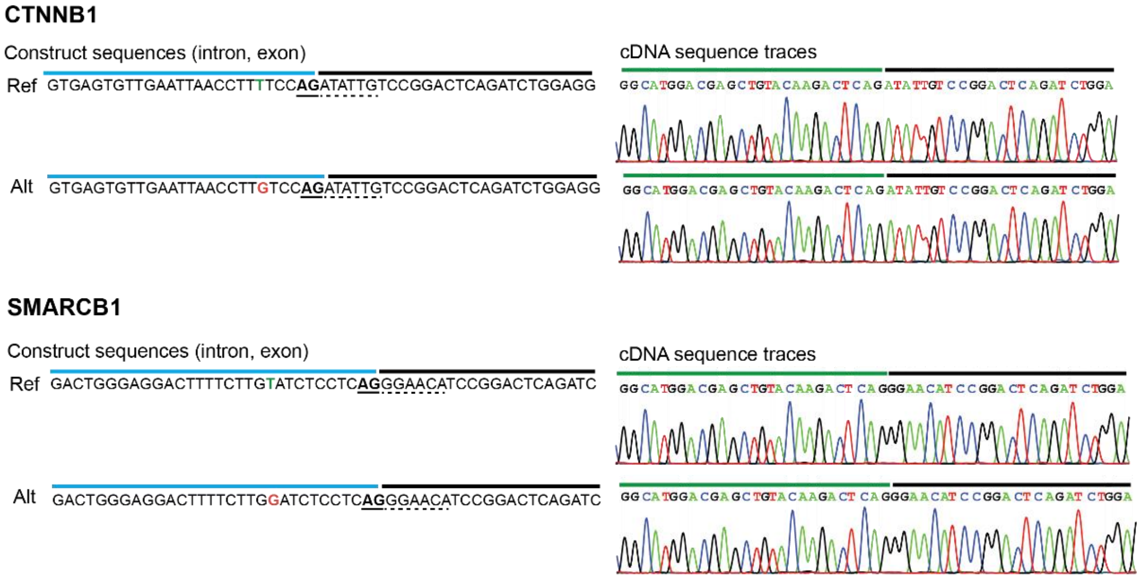

D. Validation outcomes for control variants in unaffected parents

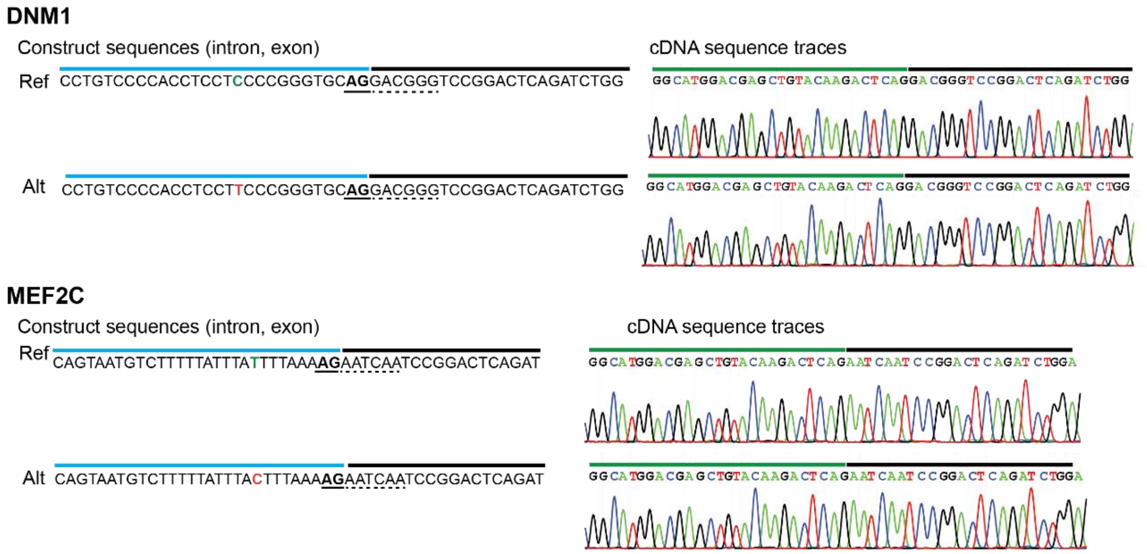

Supplemental Fig. S5 – Proportion of parental variants in high pLI gene by pathogenicity score

Proportion of variants (with 95% CI) in 13,750 unaffected parents of DDD probands which fall within genes with high pLI (>0.9) for pathogenicity score brackets (least to most severe), with Spearman correlation coefficient.

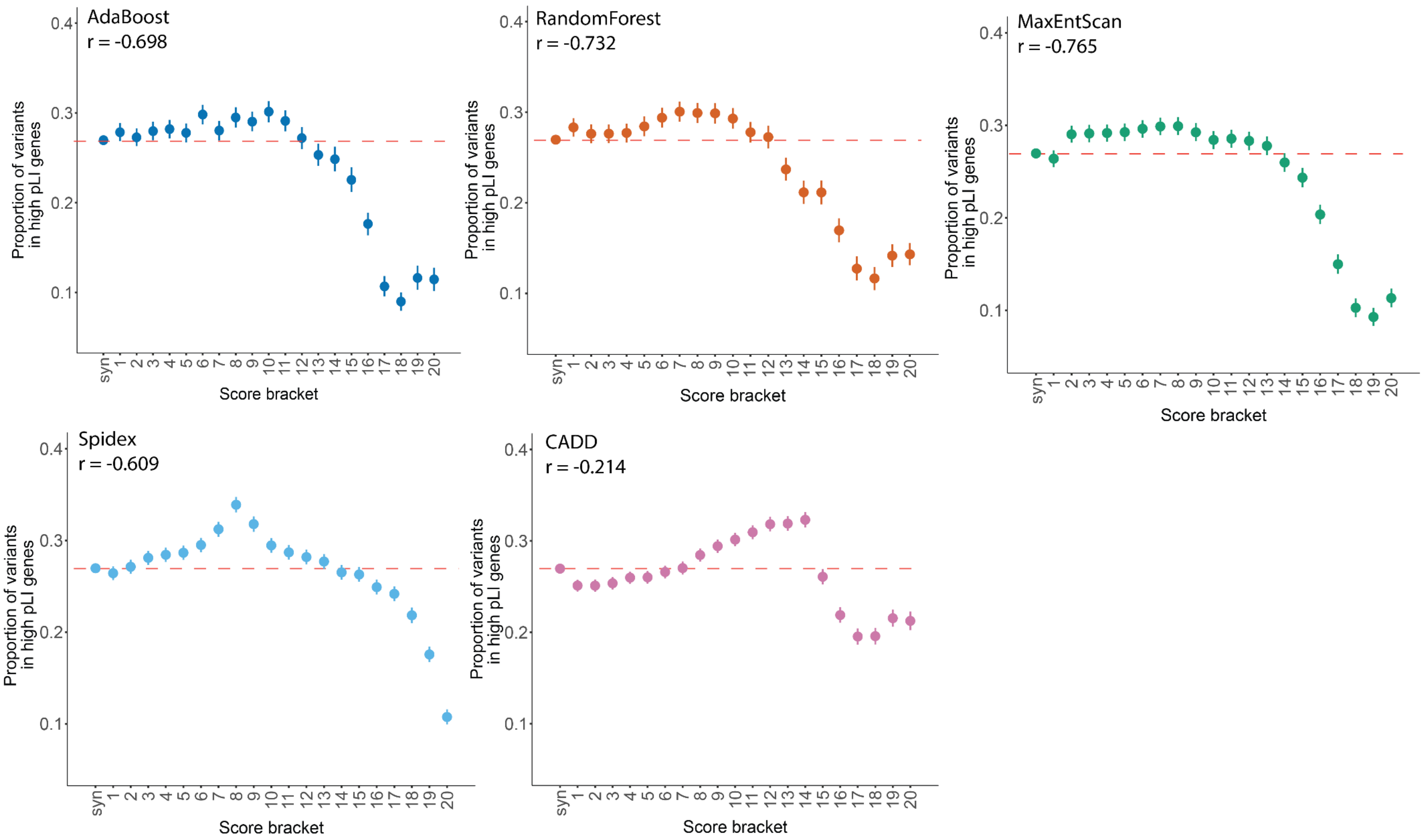
AdaBoost
r = -0.698
RandomForest
r = -0.732
MaxEntScan
r = -0.765
Spidex
r = -0.609
CADD
r = -0.214
Proportion of variants in high pLI genes
Score bracket
syn 1 2 3 4 5 6 7 8 9 10 11 12 13 14 15 16 17 18 19 20
0.1 0.2 0.3 0.4

Supplemental Fig. S6 – Mutability adjusted proportion of singletons by pathogenicity score, excluding canonical splice sites

Mutability adjusted proportion of singletons (MAPS) with 95% CI calculated for pathogenicity score brackets (least to most severe), excluding canonical dinucleotide positions, in 13,750 unaffected parents from the DDD project, with Spearman correlation coefficient.

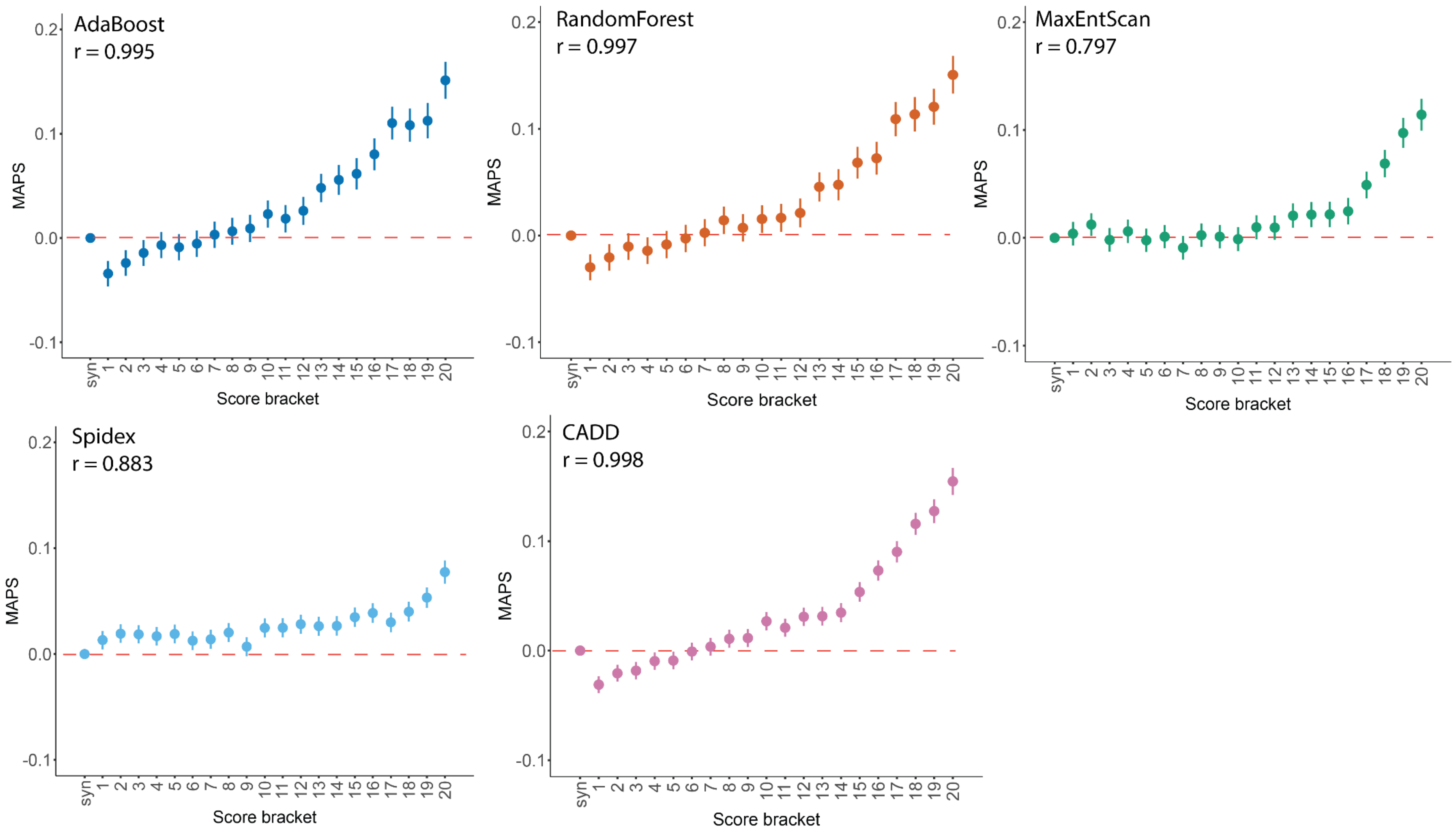
AdaBoost
r = 0.995
RandomForest
r = 0.997
MaxEntScan
r = 0.797
Spidex
r = 0.883
CADD
r = 0.998
MAPS
Score bracket
0.2
0.1
0.0
-0.1
syn 1 2 3 4 5 6 7 8 9 10 11 12 13 14 15 16 17 18 19 20

Supplemental Fig. S7 – Proportion of parental variants in high pLI gene by pathogenicity score, excluding canonical splice sites

Proportion (with 95% CI) of variants in 13,750 unaffected parents of DDD probands which fall within genes with high pLI (>0.9) for pathogenicity score brackets (least to most severe), excluding canonical dinucleotide positions, with Spearman correlation coefficient.

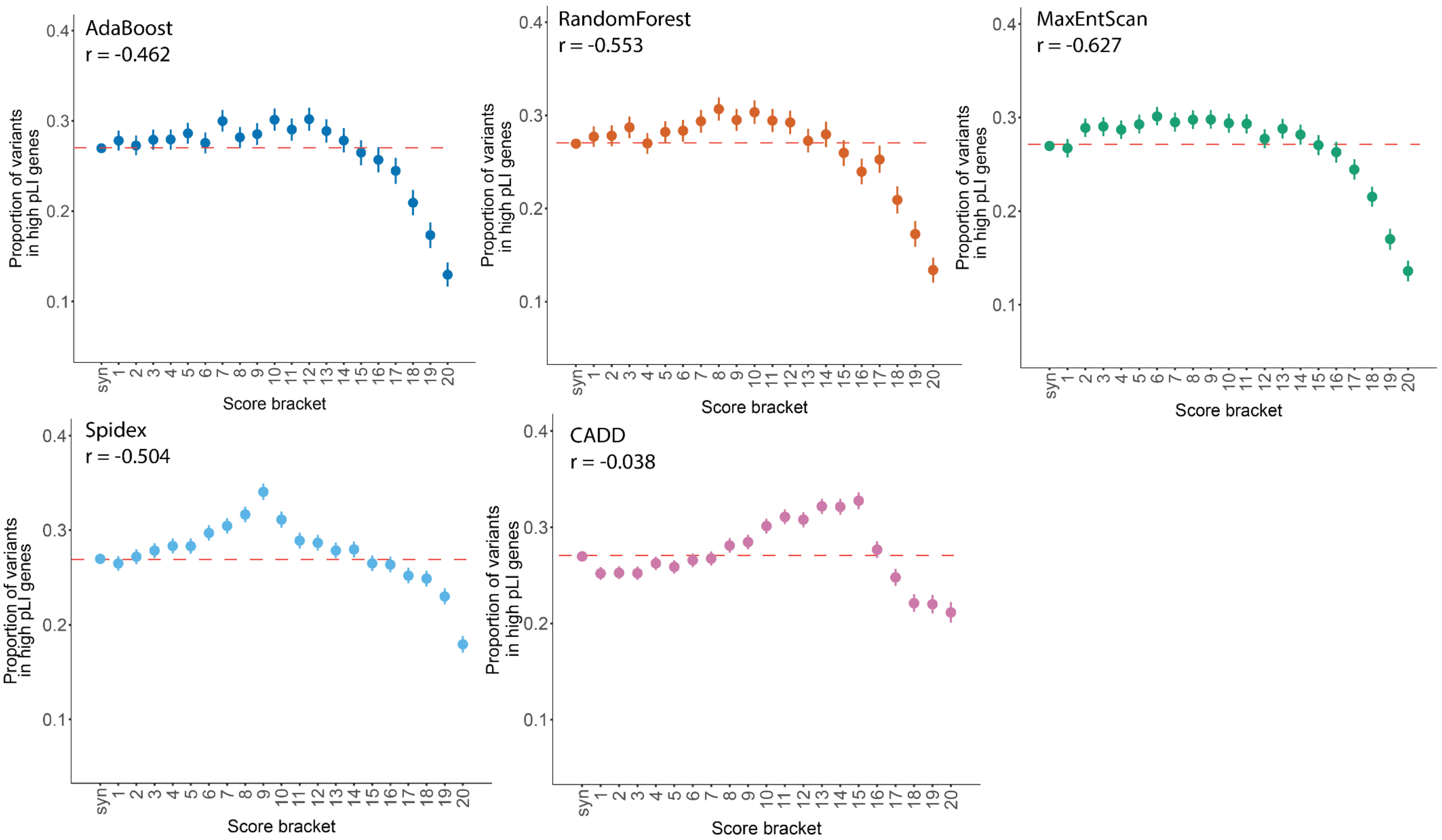
AdaBoost
r = -0.462
RandomForest
r = -0.553
MaxEntScan
r = -0.627
Spidex
r = -0.504
CADD
r = -0.038
Proportion of variants in high pLI genes
Score bracket
0.1
0.2
0.3
0.4
syn
1
2
3
4
5
6
7
8
9
10
11
12
13
14
15
16
17
18
19
20

Supplemental Table S5 – Details of variants selected for validation assay (hg19 coordinates)

Gene of interest	Variant	Intron	Splice annotation	Strand	Reason for validation	Genomic region assayed	Minigene assay outcome
GLI3	7:42063221_G/C	9-10	acc -14	-	PolyPy likely pathogenic	7:42063202-42063393	13bp intron retention
MEF2C	5:88025173_A/C	8-9	acc -9	-	PolyPy likely pathogenic	5:88025159-88025327	8bp intron retention
CHD7	8:61763045_G/A	25-26	acc -7	+	PolyPy likely pathogenic	8:61762849-61763057	5bp intron retention (mixed product)
MBD5	2:149221493_G/C	8-9	don+5	+	Don+5 likely pathogenic	2:149221483-149221651	12bp intron retention (mixed product)
CREBBP	16:3819367_C/T	14-15	acc -13	-	PolyPy likely pathogenic	16:3819349-3819526	11bp intron retention
SMARCB1	22:24143120_T/G	3-4	acc -11	+	PolyPy likely pathogenic	22:24142916-24143136	No effect on splicing
DNM1	9:130988306_G/A	9-10	acc -8	+	PolyPy likely pathogenic	9:130988199-130988319	6bp intron retention
CTNNB1	3:41266439_T/G	3-4	acc -6	+	PolyPy uncertain significance	3:41266294-41266450	No effect on splicing
MEF2C control	5:88025173_A/G (paternal)	8-9	acc -9	-	Negative control	5:88025159-88025327	No effect on splicing
DNM1 control	9:130988302_C/T (maternal)	9-10	acc -12	+	Negative control	9:130988199-130988319	No effect on splicing

Supplemental Table S6 – Primers used to amplify region of interest from patient DNA

Gene of interest	Variant	FWD primer	REV primer
**GLI3**	7:42063221_G/C	TGTCTCATAGATGACTTCAG	TAAAGACCTGATTTGATTTATTCTC
**MEF2C**	5:88025173_A/C	TTCCTTGTCCTGGTAAAGTAGGA	CCTACTCATTTGCTCTCTGCTG
**CHD7**	8:61763045_G/A	GATCTGGGGAAAAATAGGGTCAGAAATC	CACCACCATCTGCTAGCATGTC
**MBD5**	2:149221493_G/C	TCCTGGAGCTGCTGTGAA	GCAGAGTATTTGTATGTACTAATTAGTGTTTATTC
**CREBBP**	16:3819367_C/T	GGAATTGGTTTCTGCGCTGG	GCACCCGTGTTCCTACCG
**SMARCB1**	22:24143120_T/G	CTCAGTCTCTCCTCCTTGCT	GCATCTAAGTGGTGGGAGC
**DNM1**	9:130988306_G/A	CCATACCTATGGAGCCCAGG	CTGATGGTGGCTGTGAGCTC
**CTNNB1**	3:41266439_T/G	GCCTTACTGAAAGTCAGAATGC	GTCAGTTCAGGGATTGCACG
**MEF2C control**	5:88025173_A/G (paternal)	TTCCTTGTCCTGGTAAAGTAGGA	CCTACTCATTTGCTCTCTGCTG
**DNM1 control**	9:130988302_C/T (maternal)	CCATACCTATGGAGCCCAGG	CTGATGGTGGCTGTGAGCTC

Supplemental Table S7 - Primers to PCR amplify ref and alt intron with Gibson overhangs

NNN = Homology to vector backbone for Gibson Assembly mediated cloning.

NNN = Homology to GOI intron

NNN = Homology to exon flanking GOI intron

Gene of interest	FWD primer	REV primer
GLI3 – REF	CTAGGCCCCAGGATAGGTACCTCTCAGAGTGTATTTGGTAAATCTGAAAATATG	CCTCCAGATCTGAGTCCGGACTGTTCCTGAAAGAAGAGGGTGG
GLI3 – ALT	CTAGGCCCCAGGATAGGTACCTCTCAGAGTGTATTTGGTAAATCTGAAAATATG	CCTCCAGATCTGAGTCCGGACTGTTCCTGAAAGAAGAGGCTGG
MEF2C – REF	CTAGGCCCCAGGATAGGTACGTAAATGATAGGGTTGGCA	CCTCCAGATCTGAGTCCGGATTGATTCTTTTAAAATAAATAAAAAGACATTA
MEF2C – ALT	CTAGGCCCCAGGATAGGTACGTAAATGATAGGGTTGGCA	CCTCCAGATCTGAGTCCGGATTGATTCTTTTAAACTAAATAAAAAGACATTA
CHD7 – REF	TAGGCCCCAGGATAGGTACGAAGTGTAACTTGGCTCCCAGTA	CCTCCAGATCTGAGTCCGGACATAGCCTGTGCCGTGC
CHD7 – ALT	TAGGCCCCAGGATAGGTACGAAGTGTAACTTGGCTCCCAGTA	CCTCCAGATCTGAGTCCGGACATAGCCTGTGCTGTGC
MBD5 – REF	GGGGTCCCCAAATAGGTACCCAGAACTAGTTTGCTATTTGTAATAAAATTACAGG	GCATGGACGAGCTGTACAAGGAACAAGTATGTAATATGGTGAAAGGTTCA
MBD5 – ALT	GGGGTCCCCAAATAGGTACCCAGAACTAGTTTGCTATTTGTAATAAAATTACAGG	GCATGGACGAGCTGTACAAGGAACAAGTATCTAATATGGTGAAAGGTTCA
CREBBP – REF	CTAGGCCCCAGGATAGGTACCCCTCTAGAACTCATTCCTACTTTAACCCCTG	CCTCCAGATCTGAGTCCGGAGGAAAGCTGTGAAAAAACCGAAAGC
CREBBP – ALT	CTAGGCCCCAGGATAGGTACCCCTCTAGAACTCATTCCTACTTTAACCCCTG	CCTCCAGATCTGAGTCCGGAGGAAAGCTGTGAAAAAACTGAAAGC
SMARCB1 – REF	CTAGGCCCCAGGATAGGTACGTGCCCCTACGTACCCTT	CCTCCAGATCTGAGTCCGGATGTTCCCTGAGGAGATACAAGAAAAG
SMARCB1 – ALT	CTAGGCCCCAGGATAGGTACGTGCCCCTACGTACCCTT	CCTCCAGATCTGAGTCCGGATGTTCCCTGAGGAGATCCAAGAAAAG
DNM1 – REF	TAGGCCCCAGGATAGGTACGTGCCCGTGCCATCCTCTCCAC	CCTCCAGATCTGAGTCCGGACCCGTCCTGCACCCGGG
DNM1 – ALT	TAGGCCCCAGGATAGGTACGTGCCCGTGCCATCCTCTCCAC	CCTCCAGATCTGAGTCCGGACCCGTCCTGCACCTGGG
CTNNB1 – REF	TAGGCCCCAGGATAGGTACGAGAACTAAAAAGTTAGTGTATAATAG	CCTCCAGATCTGAGTCCGGACAATATCTGGAAAAGGTTAATTC
CTNNB1 – ALT	TAGGCCCCAGGATAGGTACGAGAACTAAAAAGTTAGTGTATAATAG	CCTCCAGATCTGAGTCCGGACAATATCTGGACAAGGTTAATTC
MEF2C – CONTROL – ALT	CTAGGCCCCAGGATAGGTACGTAAATGATAGGGTTGGCA	CCTCCAGATCTGAGTCCGGATTGATTCTTTTAAAGTAAATAAAAAGACATTA
DNM1 – CONTROL - ALT	TAGGCCCCAGGATAGGTACGTGCCCGTGCCATCCTCTCCAC	CCTCCAGATCTGAGTCCGGACCCGTCCTGCACCCGGGAAG

Supplemental Table S8 – PCR primers to amplify vector backbone

	FWD primer	REV primer
Acceptor mutations	TCCGGACTCAGATCTGGAGGC	GTACCTATCCTGGGGCCTAGCCACC
Donor mutations	GGTACCTATTTGGGGACCCC	CTTGTACAGCTCGTCCATGCC

Supplemental Table S9 - RT-PCR primers

	FWD primer	REV primer
Exon 1 – Exon 2	CTGAGCACCCAGTCCAAG	GATGCCCATGGCCTTGGA
Exon 2 – Exon 3	GACCATCACCTCCAGGGAGATC	GAGCACACACTTGCAGCTCA
Exon 1 – Exon 3	ACGAGAAGCGCGATCACAT	CGTAATACGACTCACTATAGTTCTA

Supplemental Table S10 - Sequencing primers

Exon 1 – Exon 2	ACGAGAAGCGCGATCACAT
Exon 2 – Exon 3	TGTCCGAGGGTACTAAGGC
Exon 1 – Exon 3	GAGCACACACTTGCAGCTCA
