# Supplementary material for: Pathogenicity and selective constraint on variation near splice sites
Source: Genome Res. 2019 Feb;29(2):159–70. doi: 10.1101/gr.238444.118 (PMC6360807; doi:10.1101/gr.238444.118)
Supplement: Supplemental Material [file supp_gr.238444.118_Supplemental_Table_S4.pdf]

Dominant DD genes	Recessive DD genes	Additional genes removed from non-DD class	Mode of inheritance
CRYAA	RMRP	SMCHD1	Digenic
FBN1	ABHD5	MTMR14	Digenic
CYP1B1	CRYAB	NHS	Hemizygous
RET	ALX1	PQBP1	Hemizygous
HESX1	RECQL4	ACSL4	Hemizygous
GJB3	CRYBB3	AFF2	Hemizygous
IGF1R	CYP1B1	MAOA	Hemizygous
ADAR	GFER	AP1S2	Hemizygous
MIR184	GALK1	ARHGEF6	Hemizygous
MIR96	FAM126A	ARX	Hemizygous
JAG1	LTBP2	ATRX	Hemizygous
MIR17HG	POMGNT1	BRWD3	Hemizygous
FGFR1	PRSS56	CUL4B	Hemizygous
CRYBA1	RAX	DCX	Hemizygous
CRYBA4	CTDP1	DLG3	Hemizygous
CRYBB2	STRA6	DMD	Hemizygous
COL2A1	TDRD7	FGD1	Hemizygous
EYA1	GLIS3	FMR1	Hemizygous
FTL	FOXE3	GDI1	Hemizygous
FOXC1	GJA1	GRIA3	Hemizygous
FOXE3	ACOX1	HSD17B10	Hemizygous
CRYGC	AGPS	HPRT1	Hemizygous
CRYGD	ALDH3A2	HUWE1	Hemizygous
GJA3	ALDH4A1	IDS	Hemizygous
GDF6	ALX3	IQSEC2	Hemizygous
HSF4	AP4M1	IL1RAPL1	Hemizygous
SIX6	ARG1	KIAA2022	Hemizygous
MITF	ARL6	KDM5C	Hemizygous
MYH9	SMPD1	RPS6KA3	Hemizygous
DMPK	AGA	L1CAM	Hemizygous
BFSP2	ATR	LAMP2	Hemizygous
PITX2	TAT	MAGT1	Hemizygous
SNX3	B4GALT7	MAMLD1	Hemizygous
SOX2	BBS10	MED12	Hemizygous
TMEM114	BBS12	PLP1	Hemizygous
ZEB2	BBS1	NLGN4X	Hemizygous
CRYAB	BBS2	OPHN1	Hemizygous
GJA1	BBS4	PAK3	Hemizygous
MYOC	BBS5	PGK1	Hemizygous
CHRNA4	BBS7	PHF6	Hemizygous
BPIFB6	CC2D1A	PHF8	Hemizygous
BRAF	CA2	PRPS1	Hemizygous
CDH15	CA8	RAB39B	Hemizygous
CREBBP	COA5	UPF3B	Hemizygous
HSPD1	CBS	ATP6AP2	Hemizygous
CIC	CCBE1	FTSJ1	Hemizygous

DEAF1	CEP152	SLC6A8	Hemizygous
DYNC1H1	CEP290	SLC9A6	Hemizygous
EHMT1	CENPJ	SMS	Hemizygous
EP300	CLN8	SRPX2	Hemizygous
EXT1	CNTNAP2	SYP	Hemizygous
FBXO25	COX10	TSPAN7	Hemizygous
FBXW4	COX15	ZDHHC9	Hemizygous
FGFR2	CPS1	ZNF711	Hemizygous
FGFR3	CRBN	CASK	Hemizygous
FOXF1	COX6B1	FLNA	Hemizygous
FOXG1	DAG1	MECP2	Hemizygous
FOXP1	DCAF17	NSDHL	Hemizygous
GATA4	GAD1	OFD1	Hemizygous
GLMN	DHCR7	SOX3	Hemizygous
SLC2A1	DLL3	GPC3	Hemizygous
HDAC4	DYM	FRMD7	Hemizygous
HNF1B	EIF2AK3	CHRDL1	Hemizygous
HOXD13	ERCC2	PIGA	Hemizygous
TCF4	ERCC3	RBM10	Hemizygous
KCNC3	ERCC5	ATP7A	Hemizygous
KCNQ2	ERCC6	SRY	Hemizygous
KIRREL3	ERCC8	TRAPPC2	Hemizygous
KRIT1	ESCO2	FHL1	Hemizygous
PAFAH1B1	FKRP	NDP	Hemizygous
LMNA	FKTN	TIMM8A	Hemizygous
MED13L	FRAS1	EDA	Hemizygous
MEF2C	FREM2	ABCB7	Hemizygous
MAPK10	WDPCP	ABCD1	Hemizygous
MLL2	FTCD	AGTR2	Hemizygous
MNX1	SLC35C1	AIFM1	Hemizygous
MAP2K1	GALE	AR	Hemizygous
MAP2K2	GALT	ARHGEF9	Hemizygous
MYH3	GAMT	ARSE	Hemizygous
MYH6	GATM	CHM	Hemizygous
NF1	GCH1	DKC1	Hemizygous
NIPBL	GDF5	FANCB	Hemizygous
NKX2-5	GLUL	FOXP3	Hemizygous
NOG	GNPAT	GK	Hemizygous
NSD1	GNPTAB	MID1	Hemizygous
TP63	GNPTG	NAA10	Hemizygous
PAX8	GRIK2	NDUFA1	Hemizygous
PDCD10	HPD	NLGN3	Hemizygous
PTEN	UROC1	OTC	Hemizygous
RAI1	HOXA1	PTCHD1	Hemizygous
RASA1	HYLS1	SLC16A2	Hemizygous
HRAS	IDUA	STS	Hemizygous
KRAS	IGF1	SYN1	Hemizygous
SALL1	CHUK	TAZ	Hemizygous
SATB2	INPP5E	TBX22	Hemizygous
SETBP1	KCNJ10	UBE2A	Hemizygous

SHANK2	KIAA1279	ZIC3	Hemizygous
SLC9A9	KCTD7	IGBP1	Hemizygous
SMAD4	L2HGDH	OCRL	Hemizygous
SMC3	LARGE	NYX	Hemizygous
SPTAN1	LFNG	CCDC22	Hemizygous
STXBP1	LRP4	GSPT2	Hemizygous
SYNGAP1	MAN2B1	MTM1	Hemizygous
TUBA1A	MANBA	HCFC1	Hemizygous
TUBB2B	MCPH1	IGSF1	Hemizygous
TBX20	MESP2	ZC4H2	Hemizygous
TBX5	MTR	KIF4A	Hemizygous
TGFBR1	MGAT2	HDAC8	Hemizygous
TLL1	MKKS	ALG13	Hemizygous
TRPS1	MKS1	BCAP31	Hemizygous
TSC1	MLC1	USP9X	Hemizygous
TSC2	MMADHC	CNKSR2	Hemizygous
YY1	MTHFR	KANK1	Imprinted
VANGL1	MYO5A	KCNK9	Imprinted
ZNF599	NAGA	CDKN1C	Imprinted
GCH1	PRSS12	IGF2	Imprinted
GDF5	NEU1	UBE3A	Imprinted
HPD	NTRK1	MAGEL2	Imprinted
PTCH1	NUP62	MTTP	Mitochondrial
KIT	SLC25A15	AKT1	Mosaic
SMARCB1	ALDH18A1	PIK3CA	Mosaic
TSHR	SLC46A1	AKT3	Mosaic
GNAS	PEPD	SLC35A2	Mosaic
SMARCA2	PEX10	SRGAP3	Uncertain
GATA6	PEX13	ZDHHC15	Uncertain
WT1	PEX1	AXIN1	Uncertain
ACTB	PEX26	BCOR	X-linked domina
ACTG1	PEX2	CDKL5	X-linked domina
GATA2	PEX5	SMC1A	X-linked domina
ASXL1	PEX7	KDM6A	X-linked domina
NOTCH2	PAH	EBP	X-linked domina
GRIN2A	PIGV	HCCS	X-linked domina
GRIN2B	POMT1	PORCN	X-linked domina
KAT6B	POMT2	COX7B	X-linked domina
RAD51	CTSA	WDR45	X-linked domina
SRCAP	BBS9	PCDH19	X-linked over-do
KIF11	ADSL	EFNB1	X-linked over-do
EFTUD2	ATIC		
ABCB6	PC		
COL4A2	RBM28		
COL4A1	SLC12A6		
EZH2	SLC17A5		
NRXN1	SLC4A4		
NRXN2	SC5D		
NRXN3	SCO1		
PRRT2	SIL1		

TAB2
GJC2
KRT74
PTHLH
SMARCA4
KBTBD13
MAP3K1
KLF1
WDR11
NR5A1
SPTLC2
NFIX
CBL
KIF22
TSHZ1
CDON
ANKRD11
SPECC1L
ARHGAP31
ANKRD26
COL11A1
KCNMA1
FGF10
ACVR1
ZNF750
SHANK3
SOS1
RNF135
RAF1
SPRED1
ACTA2
FAM58A
AFG3L2
TRPV4
IRF6
TEK
THAP1
SOX9
SHOC2
THRA
TBX4
KIF1A
PRRX1
IHH
SLC4A1
AMER1
ANKH
ANO5
CHD7
COL3A1
SOBP
TUBA8
TBCE
GTF2H5
TRAPPC9
TRIM32
TTC8
TUSC3
UBR1
UMPS
VLDLR
WDR62
WNT10B
WNT7A
XPNPEP3
ZBTB16
ZFYVE26
HSPD1
LMNA
ACBD6
ADK
ADRA2B
ASCC3
ASCL1
ARL14EP
C12orf57
TTI2
RABL6
CACNA1G
CAPN10
CASP2
CCNA2
CNKSR1
COQ5
EEF1B2
ELP2
ENTPD1
ERLIN2
FASN
FRY
GON4L
HIST1H4B
HIST3H3
INPP4A
KDM5A
KDM6B
KIF7
LAMA1
LARP7
LINS

COL9A1
COL9A2
COL9A3
COL10A1
COL11A2
COMP
DSPP
FLNB
FOXL2
LDB3
LMX1B
LRP5
LEMD3
MATN3
MMP13
MYH8
IKBKG
ACAN
PTH1R
RUNX2
SIX1
STAT1
TGFB1
COL4A3
DFNA5
EDNRB
EYA4
FGF9
GJB2
MYCN
PAX3
PTPN11
SIX5
TECTA
DYRK1A
GABRB3
PITX1
TFAP2A
WNT4
ACVR2B
ALX4
ATP1A3
CFC1
CHRNB2
COL1A1
CRELD1
CRX
ELN
EMX2
FBN2
MAN1B1
NDST1
PARP1
PECR
POLR3B
PRMT10
PRRT2
RALGDS
RGS7
SCAPER
SLC31A1
TAF2
TMEM135
TRMT1
UBR7
WDR45B
ZBTB40
ZCCHC8
ZNF526
ATM
PALB2
BRIP1
BUB1B
DDB2
ERCC4
NBN
SBDS
XPA
XPC
FANCG
FANCF
FANCE
FANCD2
FANCC
PMS2
FH
BRCA2
BLM
TSHR
CTC1
PNPLA1
RBM8A
CEP41
MEGF10
CEP57
ORC1
ORC4
ORC6
CDC6
COLEC11

FLT4	MASP1
FOXC2	ACP5
FOXP2	CCDC39
GABRA1	CCDC40
GFAP	SDCCAG8
GLI2	NUBPL
GLI3	TMEM216
GLUD1	RAD51C
HNF4A	PNKP
ITGA6	FKBP14
ITPR1	AGK
LEFTY2	GPR179
LHX4	DDOST
MSX1	ENPP1
MSX2	ABCC6
NKX2-1	SLC33A1
NODAL	RIPK4
NOTCH1	C8orf37
PAX9	NRXN1
PDHA1	COX14
RANBP2	CHSY1
ROR2	IFT122
RPS19	HPSE2
SALL4	SERPINB6
SCN1A	POMP
SCN2A	PTPRQ
SCN4A	ITCH
SIX3	FLVCR2
SMAD3	TPRN
SUMO1	SLC52A3
TBX1	GRXCR1
TBX3	SH3PXD2B
TFAP2B	AIMP1
TGFBR2	PLEC
TGIF1	PSMB8
TWIST1	JAM3
WNT5A	FLVCR1
ZIC2	MED17
KCNE1	COL11A1
BMP4	SLC24A1
POLR1D	BMPER
GJA8	SCARF2
GDF3	TBC1D24
OTX2	FAM161A
PAX2	WDR35
PAX6	PTPN14
PITX3	PDE6G
SHH	PVRL4
MAF	HSD17B4
SOX10	TWIST2

TCOF1	C12orf65
EXT2	TMEM237
PHOX2B	ELOVL4
CD96	SMOC2
GDF1	WDR19
SOX17	POLR3A
ZEB1	NFU1
NRAS	BOLA3
MAFB	CSTA
SCN8A	ST3GAL3
LOXHD1	PXDN
TRPV3	KIF1A
ARID1B	IGFBP7
PDE4D	DOCK6
ARID1A	IL11
SMARCE1	CCDC8
GUCY2C	CUL7
SHANK1	BLOC1S6
SF3B4	FYCO1
PLCB4	NDE1
GNAI3	CLN6
KANSL1	IMPAD1
RAD21	FAM20A
ABCC9	BANF1
PIK3R2	LIPN
TGFB2	ILDR1
PHF21A	DHFR
MBD5	IHH
NR1I3	NEK1
CARD14	GJC2
IFITM5	CFL2
MLL	TNFRSF13B
CCDC78	SECISBP2
RBPJ	SLC4A1
DIP2B	MMACHC
YWHAE	FLG
KLF8	SLC2A10
ZFPM2	MPV17
SKI	PLA2G6
GATAD2B	SLC4A11
PIEZO2	DFNB59
PLCG2	SLC6A5
KCNT1	RNASEH2B
PACS1	RNASEH2C
CHD8	RNASEH2A
TBR1	TREX1
TBL1XR1	ATP13A2
SNRPE	SEC23A
AUTS2	RSPO4
STIM1	DMP1

TCF12	PLCE1
ERF	PNPLA2
KCTD1	SYNE1
DEPDC5	HAX1
TUBB4A	LEPRE1
PRKAR1A	SPG11
KIF5C	EOMES
KIF2A	IFT80
BICD2	LRP2
FAM111A	GLIS2
PDGFRB	GLE1
CHD2	PCNT
SCN1B	AK2
POLD1	TBXAS1
DSTYK	TMPRSS6
CACNA1A	HPGD
GRIN1	MYO5B
HNRNPU	TMEM70
MTOR	LRTOMT
NEDD4L	TAC3
CTNNB1	TACR3
MTF1	SLC25A38
ZMYM6	SDHAF1
MYT1L	RNASET2
PHIP	TACO1
WAC	SEC23B
ASH1L	PYCR1
CAMTA1	FAM134B
CACNA1C	VIPAS39
GNAO1	ARSB
VIP	OBSL1
CAPRIN1	GLB1
CTCF	GUSB
PIK3R1	CTSD
RIT1	CLN3
TM4SF20	CLN5
RARB	NAGLU
SMAD2	CTNS
NAA15	FUCA1
SCN11A	GALNS
C6orf70	GBA
QKI	GNS
PTDSS1	HEXA
LRP2	HEXB
GRHL3	HGSNAT
PUF60	HYAL1
SCRIB	PEX12
EEF1A2	PEX14
CLCN4	PEX16
KCNH5	PEX19

DNM1
SNRPB
YAP1
NR2F1
EDN1
RRAS
MAB21L2
NR2F2
SETD5
TUBB2A
AHDC1
IFIH1
ADNP
DNMT3A
CCND2
HCN1
ZSWIM6
ZBTB20
ASXL3
SOX11
ZNF713
CACNA1D
NOTCH3
COL4A3BP
PPP2R5D
POGZ
PPP2R1A
DDX3X
BCL11A
PURA
TRIO
PCGF2
NFIA
TGFB3
KCNH1
ALDH18A1
CHAMP1
SHOX

PEX3
PEX6
PPT1
GM2A
SGSH
TPP1
ALG12
ANO5
NPR2
CHST3
COL9A1
COL11A2
DDR2
DOCK8
FREM1
RAB18
RAB3GAP1
DYNC2H1
EVC
FZD6
GPC6
IFT43
EVC2
LBR
LRP5
MMP13
NKX3-2
NPHP1
NPHP3
NPHP4
PAPSS2
HSPG2
ACAN
PTH1R
SLC26A2
SLC35D1
SLC39A13
STAT1
TTN
TRIP11
AFG3L2
FLNB
ALDH5A1
AUH
BCS1L
BTD
DDC
ETFA
ETFB
ETFDH

FAH
LMBRD1
MAT1A
MCEE
MMAA
MMAB
MUT
OXCT1
PSAP
VDR
RYR1
ADCK3
UGT1A1
ALMS1
ATP6V1B1
BSND
CD151
CDH23
CISD2
CLCNKA
CLCNKB
CLDN14
COL4A3
COL4A4
ESPN
ESRRB
FGF3
GJB2
GJB6
GPSM2
HGF
KCNE1
KCNQ1
LHFPL5
LHX3
LOXHD1
MARVELD2
MGP
MSRB3
MYO15A
MYO7A
OTOA
OTOF
PLOD2
PLOD3
AP4B1
AP4E1
AP4S1
CHRNA1
CHST14

COQ9
DPM3
FOLR1
FTO
MRE11A
NPHS1
RAD50
RFT1
SLC27A4
STIL
VRK1
WNT4
AAAS
AASS
ABCB11
ACADM
ACADS
ACADVL
ACAT1
ADA
AGL
AGXT
AIRE
ALAD
ALDH7A1
ALDOA
ALDOB
ALG1
ALG11
ALG2
ALG3
ALG6
ALG8
ALG9
ALPL
ALX4
APTX
ARFGEF2
ARSA
ASAH1
ASL
ASPA
ASPM
ASS1
ATP8B1
BCKDHA
BCKDHB
BIN1
BMPR1B
CDH3

CDK5RAP2
CIRH1A
CLCN7
COG1
COG4
COG5
COG7
COG8
COL18A1
COQ2
CSTB
CTSK
CYB5R3
DARS2
DBT
DHCR24
DLAT
DLD
DOLK
DPAGT1
DPM1
ERCC1
ETHE1
FANCI
FANCL
FANCM
FBP1
FIG4
FOXE1
FOXN1
GAA
GALC
GCDH
GCSH
GFM1
GHR
GLDC
GPR56
HADH
HLCS
HMGCL
HMGCS2
HPS1
HR
IGHMBP2
ITGA7
IVD
JAK3
KARS
KCNJ11

LAMA2
LRAT
LRPPRC
LYST
MC2R
MCCC1
MCCC2
MCOLN1
MFSD8
MLYCD
MOCS1
MOCS2
MOGS
MPDU1
MPI
NAGS
NDUFAF2
NDUFS1
NDUFS4
NDUFS7
NDUFS8
NDUFV1
NEK8
NHP2
NOP10
PDHX
PDSS1
PDSS2
PKHD1
PLCB1
PLOD1
PMM2
POU1F1
PROP1
PTF1A
PTH
PTS
PVRL1
PYGL
QDPR
RARS2
RELN
ROBO3
ROR2
RPE65
SAMHD1
SCO2
SDHA
SLC19A3
SLC22A5

SLC25A19
SLC25A20
SLC25A22
SLC2A2
SLC35A1
SLC5A5
SLC6A3
SMARCAL1
SPR
SRD5A3
ST3GAL5
STAR
SUCLG1
SUMF1
SURF1
TCTN1
TCTN2
TH
TK2
TRIM37
TSEN2
TSEN34
TSEN54
TUFM
TYR
TYRP1
UQCRB
UQCRQ
UROS
VPS13B
VPS33B
WNT3
WRAP53
ZFP57
ZMPSTE24
CRYBB1
CLDN19
CC2D2A
DHODH
POLR1C
HMX1
TMEM67
MFRP
DDX11
RPGRIP1L
RAB3GAP2
SMOC1
VSX2
FANCA
CDT1

FOXRED1
GRM6
TRPM1
ACY1
ATOH7
CAV1
ERBB3
FAM20C
HIBCH
LIG4
MED23
PIP5K1C
PREPL
RAB23
SNAP29
ST14
TTC19
MPLKIP
ACAD9
SLX4
C2orf71
ACO2
PIGL
TTC37
SKIV2L
C5orf42
ROGDI
RNF168
DNAAF3
COL9A2
NSUN2
IFT140
CEP135
GUCY2C
ISPD
UVSSA
EXOSC3
IRX5
CCDC103
LRBA
MTO1
TMEM165
PIGO
HOXB1
POC1A
TCTN3
RFX6
ITGA3
NMNAT1
PDE6H

RTTN
GTDC2
GRM1
C4orf26
MMP14
CRB1
RPGRIP1
AIPL1
KIAA0226
DNMT3B
LRIG2
EPG5
UBE3B
DIS3L2
DLX5
BRAT1
CRADD
TUBGCP6
SNIP1
HARS
CABP2
HYDIN
HEATR2
RMND1
HINT1
ABCD4
OTOGL
MEGF8
HOXC13
PNPT1
LRRC6
CHMP1A
CIB2
CRYAA
CLPP
DDHD1
CYP2U1
TECPR2
DDHD2
DHTKD1
EXPH5
TMEM5
LRIT3
CCDC114
INPPL1
ECEL1
MEOX1
ADAMTS18
GBA2
ALDH1A3

SLC24A4
B3GALNT2
TMCO1
NPHS2
DRC1
WNT1
PGAP2
EOGT
LARS2
CEP63
STAMBP
DARS
ANTXR1
PIGN
NDNL2
SPRTN
PSAT1
B3GALT6
PCCA
PCCB
PCBD1
NPC1
NPC2
NT5C3A
PHGDH
PSPH
AMT
MTRR
LAMC3
DGAT1
TTC7A
DYX1C1
AMPD2
RSPH1
DDX59
SZT2
WDR60
FBXL4
DSG1
KLHL40
GMPPB
PIK3R1
TRAPPC11
ELAC2
LRPAP1
ZMYND10
ARMC4
CYC1
RARB
GMPPA

NALCN
SPAG1
C21orf59
CCDC65
TECTA
DCHS1
FAT4
BRCA1
COASY
CSPP1
KPTN
PCYT1A
EIF4A3
IFT172
WDR34
PHC1
DNA2
SACS
EDN1
PGM3
MAB21L2
CEP83
DOCK7
PET100
ERCC6L2
PGAP3
ITGA8
XYLT1
CA5A
QARS
ASPH
SLC13A5
MICU1
CCNO
NGLY1
JAGN1
SPEG
POC1B
C2CD3
PTPRF
CACNA1D
RTEL1
PRRX1
FEZF1
APOPT1
GRHL2
CCDC151
CKAP2L
STT3A
STT3B

CLP1
NEB
PLK4
PLXND1
SNX14
LAMB1
FAR1
SHOX
RNU4ATAC

nt
nt
nt
nt
nt
nt
nt
nt
nt
minance
minance
